# Optimisation and evaluation of viral genomic sequencing of SARS-CoV-2 rapid diagnostic tests: a laboratory and cohort-based study

**DOI:** 10.1016/S2666-5247(23)00399-3

**Published:** 2024-04-12

**Authors:** Jillian S Paull, Brittany A Petros, Taylor M Brock-Fisher, Samantha A Jalbert, Victoria M Selser, Katelyn S Messer, Sabrina T Dobbins, Katherine C DeRuff, Davy Deng, Michael Springer, Pardis C Sabeti

**Affiliations:** Department of Systems Biology, Harvard Medical School, Boston, MA, USA (J S Paull AB, B A Petros PhD, S A Jalbert MLA, Prof M Springer PhD); Broad Institute of MIT and Harvard, Cambridge, MA, USA (J S Paull, B A Petros, T M Brock-Fisher MSc, K S Messer BS, S T Dobbins MS, K C DeRuff BS, D Deng MS, Prof M Springer, Prof P C Sabeti MD DPhil);Division of Health Sciences and Technology, Harvard Medical School and MIT, Cambridge, MA, USA (B A Petros); Harvard/MIT MD-PhD Program, Harvard Medical School, Boston, MA, USA (B A Petros); Howard Hughes Medical Institute, Chevy Chase, MD, USA (T M Brock-Fisher, Prof P C Sabeti); Department of Organismic and Evolutionary Biology, Harvard University, Cambridge, MA, USA (T M Brock-Fisher, Prof P C Sabeti); Montachusett Public Health Network, Fitchburg, MA, USA (V M Selser BS); Wyss Institute for Biologically Inspired Engineering, Harvard University, Boston, MA, USA (Prof M Springer); Department of Immunology and Infectious Diseases, Harvard T H Chan School of Public Health, Harvard University, Boston, MA, USA (Prof P C Sabeti)

## Abstract

**Background:**

Sequencing of SARS-CoV-2 from rapid diagnostic tests (RDTs) can bolster viral genomic surveillance efforts; however, approaches to maximise and standardise pathogen genome recovery from RDTs remain underdeveloped. We aimed to systematically optimise the elution of genetic material from RDT components and to evaluate the efficacy of RDT sequencing for outbreak investigation.

**Methods:**

In this laboratory and cohort-based study we seeded RDTs with inactivated SARS-CoV-2 to optimise the elution of genomic material from RDT lateral flow strips. We measured the effect of changes in buffer type, time in buffer, and rotation on PCR cycle threshold (Ct) value. We recruited individuals older than 18 years residing in the greater Boston area, MA, USA, from July 18 to Nov 5, 2022, via email advertising to students and staff at Harvard University, MA, USA, and via broad social media advertising. All individuals recruited were within 5 days of a positive diagnostic test for SARS-CoV-2; no other relevant exclusion criteria were applied. Each individual completed two RDTs and one PCR swab. On Dec 29, 2022, we also collected RDTs from a convenience sample of individuals who were positive for SARS-CoV-2 and associated with an outbreak at a senior housing facility in MA, USA. We extracted all returned PCR swabs and RDT components (ie, swab, strip, or buffer); samples with a Ct of less than 40 were subject to amplicon sequencing. We compared the efficacy of elution and sequencing across RDT brands and components and used RDT-derived sequences to infer transmission links within the outbreak at the senior housing facility. We conducted metagenomic sequencing of negative RDTs from symptomatic individuals living in the senior housing facility.

**Findings:**

Neither elution duration of greater than 10 min nor rotation during elution impacted viral titres. Elution in Buffer AVL (Ct=31⋅4) and Tris-EDTA Buffer (Ct=30⋅8) were equivalent (p=0⋅34); AVL outperformed elution in lysis buffer and 50% lysis buffer (Ct=40⋅0, p=0⋅0029 for both) as well as Universal Viral Transport Medium (Ct=36⋅7, p=0⋅079). Performance of RDT strips was poorer than that of matched PCR swabs (mean Ct difference 10⋅2 [SD 4⋅3], p<0⋅0001); however, RDT swabs performed similarly to PCR swabs (mean Ct difference 4⋅1 [5⋅2], p=0⋅055). No RDT brand significantly outperformed another. Across sample types, viral load predicted the viral genome assembly length. We assembled greater than 80% complete genomes from 12 of 17 RDT-derived swabs, three of 18 strips, and four of 11 residual buffers. We generated outbreak-associated SARS-CoV-2 genomes using both amplicon and metagenomic sequencing and identified multiple introductions of the virus that resulted in downstream transmission.

**Interpretation:**

RDT-derived swabs are a reasonable alternative to PCR swabs for viral genomic surveillance and outbreak investigation. RDT-derived lateral flow strips yield accurate, but significantly fewer, viral reads than matched PCR swabs. Metagenomic sequencing of negative RDTs can identify viruses that might underlie patient symptoms.

**Funding:**

The National Science Foundation, the Hertz Foundation, the National Institute of General Medical Sciences, Harvard Medical School, the Howard Hughes Medical Institute, the US Centers for Disease Control and Prevention, the Broad Institute and the National Institute of Allergy and Infectious Diseases.

## Introduction

COVID-19 has led to rapid advances in infectious disease diagnostics; in particular, direct-to-consumer, at-home SARS-CoV-2 testing has become ubiquitous. Compared with conventional PCR tests, rapid diagnostic tests (RDTs) offer prompt results with low costs. The quick turnaround of results makes RDTs an important tool for slowing disease spread, even when compared with more sensitive tests, such as PCR.^1^ Moreover, RDTs might be more effective than PCR tests at identifying individuals who are infectious.^2^ With the 2023 release of the first US Food and Drug Administration (FDA)-approved over-the-counter influenza and SARS-CoV-2 rapid test, RDTs are becoming a pillar of respiratory infectious disease management.

However, as reliance on RDTs increases, one key advantage of traditional PCR tests is lost: the ability to conduct high-quality viral genomic sequencing from the residual genetic material, thereby offering relevant epidemiological, evolutionary, and public health insights. PCR testing enables centralised storage of surplus genomic material that can be readily used for genomic sequencing. Moreover, robust protocols exist to sequence from PCR swabs; these protocols largely rely upon the same upstream processing steps needed to run PCR tests.^3^

To maintain genomic surveillance, standardised protocols to extract and sequence genetic material directly from RDTs are needed. Previous work suggests that sequencing from non-conventional sample types (eg, sequencing of SARS-CoV-2 positive wastewater samples)^4^ holds promise. Although studies have provided proof-of-concept evidence for the feasibility of sequencing from RDTs,^5,6^ protocols have not been optimised among a number of axes that might affect elution and subsequent sequence quality. Moreover, it is possible that distinct RDT brands and components (eg, swabs, residual buffer, or strips) produce sequencing data of varying quality. Additionally, no studies have directly compared RDT sequences to those generated from person-matched PCR swabs. In this study, we aim to develop, evaluate, and implement a protocol for genomic sequencing of SARS-CoV-2 from RDTs employing antigen capture with lateral flow technology.

## Methods

### Study design and samples

**Figure 1:**
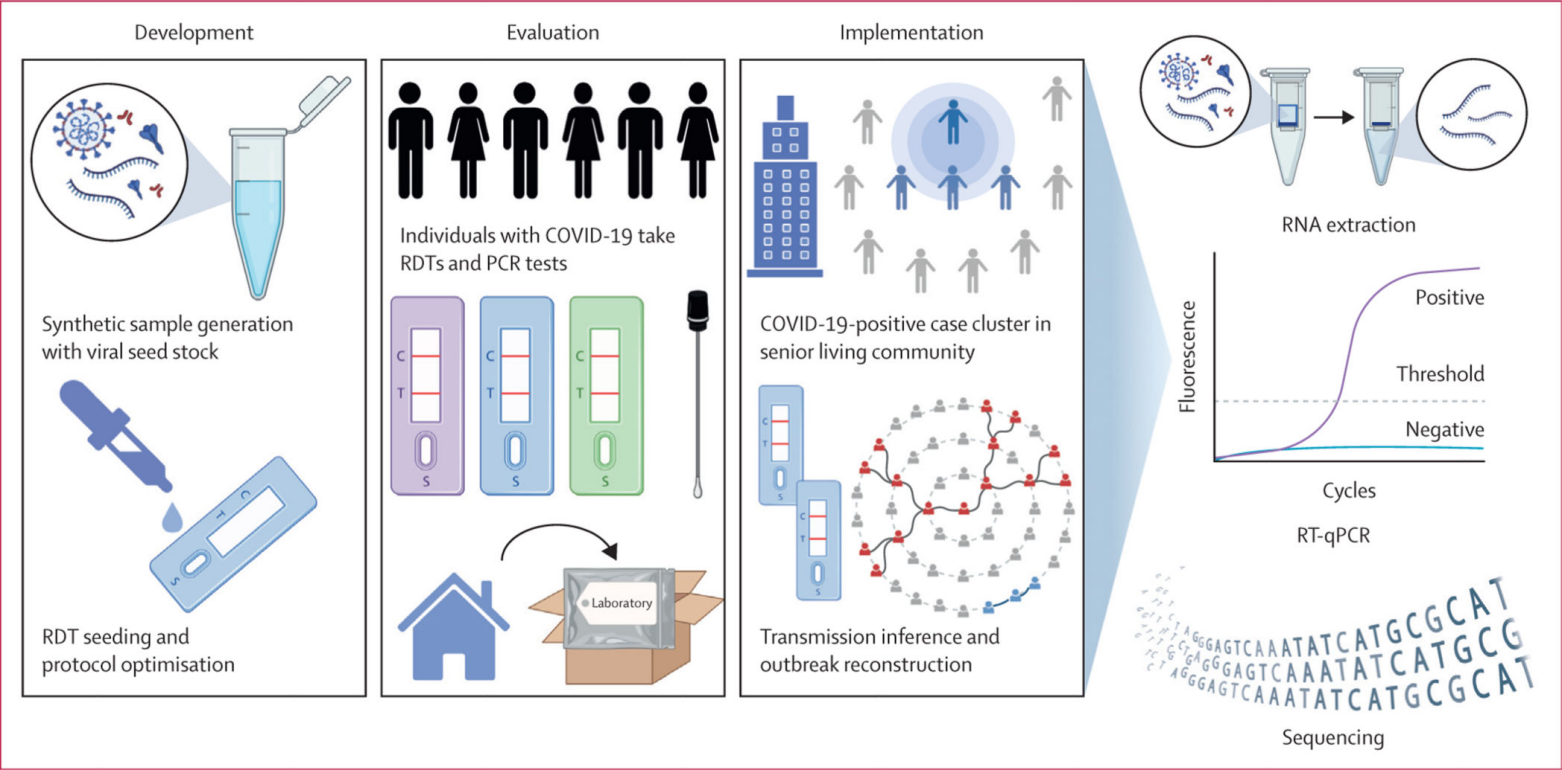
Study overview A protocol to sequence SARS-CoV-2 from RDT strips was developed using synthetic samples to seed rapid tests. The protocol was evaluated across three RDT brands relative to the gold standard (PCR swabs) within and across individuals. Sequencing of RDTs was implemented in the investigation of an outbreak in a senior living apartment building. Transmission inference and outbreak reconstruction were performed with genomes generated from residual RDT components. Figure created with BioRender.com. RDT=rapid diagnostic test. RT-qPCR=RT-quantitative PCR.

This laboratory and cohort-based study consisted of three parts: (1) the development of a protocol for elution of SARS-CoV-2 genetic material from RDT strips, and the application of this protocol to SARS-CoV-2-positive clinical samples; (2) for the evaluation of RDT brands and components; and (3) for outbreak investigation and transmission inference (figure 1).

The three RDT brands used in this study (Abbott Binax-NOW, Quidel QuickVue, and iHealth; all reagent, kit, and equipment manufacturer details are shown in appendix 1 p 13) represent different testing strategies. All strategies apply an anterior nares swab to a moistened strip (Binax-NOW; Lake Forest, IL, USA) or buffer tube; for the buffer tube, the strip is placed within the buffer (QuickVue; San Diego, CA, USA) or buffer is added to the strip (iHealth; San Jose, CA, USA).

In cohort 1, we enrolled individuals positive for SARS-CoV-2 under the Harvard Longwood Campus (Boston, MA, USA) institutional review board (IRB) protocol 20–1877, with the work covered by an exempt determination (EX-7295) at the Broad Institute (Cambridge, MA, USA), indicating that the research does not require approval by a second IRB. All participants provided electronic informed consent. Participants older than 18 years in the greater Boston area were recruited from July 18 to Nov 5, 2022, via email advertisement to students and staff at Harvard University, MA, USA, and social media advertisement to target a broader audience. Individuals were included if 5 or less days had passed since their COVID-19 diagnostic test; no other exclusion criteria were applied. Participants were sent two RDTs (iHealth, BinaxNOW, or QuickVue), a RHINOstic polypropylene swab (ie, PCR swab), and instructions (appendix 1 pp 18–23). Participants were asked to return the PCR swab, RDT strips, BinaxNOW swabs, and QuickVue buffer tubes. We did not request iHealth or QuickVue swabs or iHealth buffer because these kit components cannot be sealed. Separate bags were included for each component, which were shipped to the Broad Institute’s Genomic Center for Infectious Diseases (Cambridge, MA, USA) overnight in a FedEx box at room temperature. Samples were stored at 4°C and were extracted within 1 week of test administration. Samples with a cycle threshold (Ct) of less than 40 were sequenced.

In cohort 2, the samples were collected by the Montachusett Public Health Network (MPHN; Fitchburg, MA, USA) as part of public health activities and were not subject to an IRB for use by MPHN. The use of these samples for sequencing and analysis at the Broad Institute was covered under protocol 1612793224, reviewed and approved by the Massachusetts Institute of Technology IRB; informed consent was not required to analyse viral genomic material from residual samples. MPHN contacted TMB-F on Dec 27, 2022, to request genomic sequencing as part of a previously established collaboration with the Broad Institute.

After nine of 164 residents of a senior housing building in MA, USA, tested positive for SARS-CoV-2 during the week of Dec 23, 2022, MPHN initiated an epidemiological investigation. MPHN interviewed residents about their social interactions within the building and potential exposures to SARS-CoV-2 (questionnaire is in appendix 1 pp 24–29). MPHN scheduled an on-site testing clinic on Dec 29, 2022, which was advertised via flyers sent to each resident and building-wide robocalls (ie, a call that delivers a pre-recorded message). At the clinic, iHealth RDTs were provided to any interested residents. 49 residents participated: eight new cases and two known cases tested positive for SARS-CoV-2, resulting in 17 total detected cases in the building. MPHN collected RDT strips, swabs, and buffer from the ten individuals with positive RDTs and from five symptomatic individuals who tested negative via RDT (two of whom previously tested positive). Samples were stored in individual bags on ice, delivered to the Broad Institute’s Genomic Center for Infectious Diseases (Cambridge, MA, USA) on the same day, and stored at 4°C for 5 days before extraction.

### Procedures

We used QuickVue tests to create synthetic samples for assay optimisation. We collected 100 μL of heat-inactivated SARS-CoV-2 (4⋅2× 10^5^ genome copies per μL), diluted 1:100 in nuclease-free water, onto the QuickVue swab and conducted the test per manufacturer’s instructions. After the test dried for 15 min, the lateral flow strip was carefully folded and placed into a 1⋅5 mL microcentrifuge tube. Gloves were changed between replicates and before and after folding the strip.

We used these strips to evaluate the effects of elution buffer, incubation time, and extraction method on viral load. In each experiment, 700 μL buffer was added to a microcentrifuge tube containing the strip. First, we tested Buffer AVL, Lysis Buffer solution (LB), 50% LB (LB50; 1:1 with nuclease-free water), Universal Viral Transport Medium, and Tris EDTA buffer pH 8 (TE). For later experiments, we used AVL.

The tube was vigorously vortexed for 15 s, incubated for a set amount of time, and then vigorously vortexed for 15 s. When varying incubation time, samples were incubated for 10 min (ie, the minimum time required for viral particle inactivation^7^), 1 h, or 24 h. When varying buffers, samples were incubated for 10 min. When testing the effect of rotation, samples were incubated for 10 min with or without rotation or for 1 h with rotation. Rotating incubations used a Digital Tube Revolver (Thermo Scientific; Waltham, MA, USA) at 40 rotations per min.

The 700 μL solution was used as input into manual nucleic acid extraction with the QIAamp Viral RNA Mini Kit (Germantown, MD, USA; appendix 1 pp 16–17). Elution efficacy was compared by calculating Ct via RT-quantitative PCR on the QuantStudio (Waltham, MA, USA; appendix 1 pp 16–17).

We assessed strip stability over time by implementing the optimised protocol (ie, incubation in AVL for 10 min without rotation) for strips left for 0–7 days at room temperature.

We compared manual and automatic extraction. We followed manufacturer’s instructions for the MagMAX mirVana Total RNA Isolation Kit (Waltham, MA, USA) with the KingFisher Flex System (KFF; Waltham, MA, USA). The standard KFF protocol adds 500 μL KFF lysis/binding mix to 200 μL sample in its original buffer. To maintain this ratio, we developed two approaches. One set of RDTs was eluted into 700 μL AVL, then 200 μL was used as sample input. A second set of RDTs was eluted into 700 μL AVL-KFF mix (200 μL AVL and 500 μL KFF lysis/binding mix), and the entire volume was used as sample input and lysis/binding mix. These two protocols are referred to as automatic (AVL Buffer) and automatic (KFF Buffer), respectively. Synthetic experiments were run with three to five replicates.

To evaluate the optimised protocol on samples from cohorts 1 and 2, RDT strips, RDT swabs, and PCR swabs were eluted as described earlier. For RDT residual buffer, AVL was added until the total amount of liquid was 700 μL. Extraction and RT-quantitative PCR approaches are detailed in appendix 1 (pp 16–17).

We assessed the elution performance of positive RDTs by comparing the Ct—a proxy for viral titre—of different components (ie, strips, swabs from RDTs and PCRs, and buffer) across and within brands. For cohort 1, we also assessed the effect of testing order on subsequently collected RDTs. Samples with Cts higher than those of the associated extraction controls were excluded from these and future analyses.

ARTIC v4.1 (Coralville, IA, USA) amplicon-based sequencing was performed^8^ on all PCR-positive samples using the NextSeq 550 (San Diego, CA, USA; appendix 1 pp 16–17). Samples were excluded from these analyses if their genome lengths were shorter than those of the associated negative controls.

We assessed the sequencing performance of RDTs by evaluating the proportion of samples yielding complete or partial genomes, mean read depth, and the ability to assign viral lineages to samples. We assessed the correlation between read depth and percent genome assembled, and the relationship between lineage assignment and viral titre. We compared the fidelity of mutation calls in sequences generated from RDTs relative to sequences generated from PCR swabs.

Unbiased RNA sequencing of the negative RDT swabs collected from symptomatic individuals in cohort 2 was conducted as previously described,^9^ with Nextera XT DNA libraries (Illumina; San Diego, CA, USA) that were subject to target enrichment (appendix 1 pp 16–17).

Using the viral-ngs v2.1.33 pipeline,^10^ reads were demultiplexed, filtered to remove adapter and contaminant sequences, and depleted of reads mapping to the human genome.

Data derived from amplicon sequencing were assembled by alignment to the reference sequence (GenBank accession number: NC_045512.2). Identification of viruses in the metagenomic data was conducted as previously described,^11^ using Kraken2^12^ and contig assembly (appendix 1 pp 16–17). Upon annotation of the *N* gene, an 11 bp deletion was noted in the genomes generated from two samples (12_R and 12_Q1_strip). Inspection of the aligned bam files revealed a true deletion of nine bp followed by two single-nucleotide variants (SNVs; 28 370A→T and 28 371G→T). We manually edited the fasta files to make the appropriate changes.

Lineages were assigned using Pango v4.2 with pango-data v1.17.^13^ Complete genomes were defined as those in which more than 80% of the genome was assembled and partial genomes were defined as those in which more than 10% of the genome was assembled.

### Statistical analysis

Python v3.9.5 and SciPy v1.10.0 were used to conduct statistical analyses, with p values of less than 0⋅05 considered significant. We imputed 40 as the Ct value for any PCR triplicate that failed to amplify, and quantified viral load as the median of three triplicate samples. For comparisons with synthetic samples or non-matched clinical samples, we used a *t* test. For comparisons of matched clinical samples, we used the Wilcoxon signed-rank test. p values were not corrected for multiple comparisons. Correlations between genome assembly length and either mean genome coverage or Ct value were assessed using Spearman rank-order correlation coefficients.

LoFreq v2.1.5 was used to identify SNVs within each sample in the first cohort.^14^ SNVs were filtered to exclude known problematic sites,^15^ including the two sites that were miscalled as deletions by the viral-ngs pipeline, and those with an allele frequency of less than 3%. The genomic regions with shared coverage of high quality (ie, no evidence of strand bias and read depth ≥100) between each RDT and its matched PCR sample were compared to determine the concordance of consensus-level (allele frequency ≥ 50%) and sub-consensus-level (allele frequency <50%) SNV calls. SNV concordance is the sum over each matched pair of the SNVs found in both the PCR and the RDT, divided by the sum of the unique set of SNVs found in either sample.

For cohort 2, we aligned RDT-swab-derived outbreak-associated genomes and the SARS-CoV-2 reference sequence (GenBank accession number: NC_045512.2) using MAFFT (v7.490).^16^ We masked known problematic sites in the SARS-CoV-2 genome^15^ and estimated a maximum-likelihood phylogeny with PhyML v3.3^17^ using a general time reversible nucleotide-substitution model and 1000 iterations of bootstrapping. We rooted the tree on the Wuhan-Hu-1 sequence and visualised it using FigTree v1.4.4.^18^

Person-to-person transmission events were modelled with outbreaker2 (v1.1.3)^19–21^ using R (v4.2.1). Additional information regarding this modelling approach is in appendix 1 (pp 16–17).

### Role of the funding source

The funders of the study had no role in study design, data collection, data analysis, data interpretation, or writing of the report.

## Results

**Figure 2:**
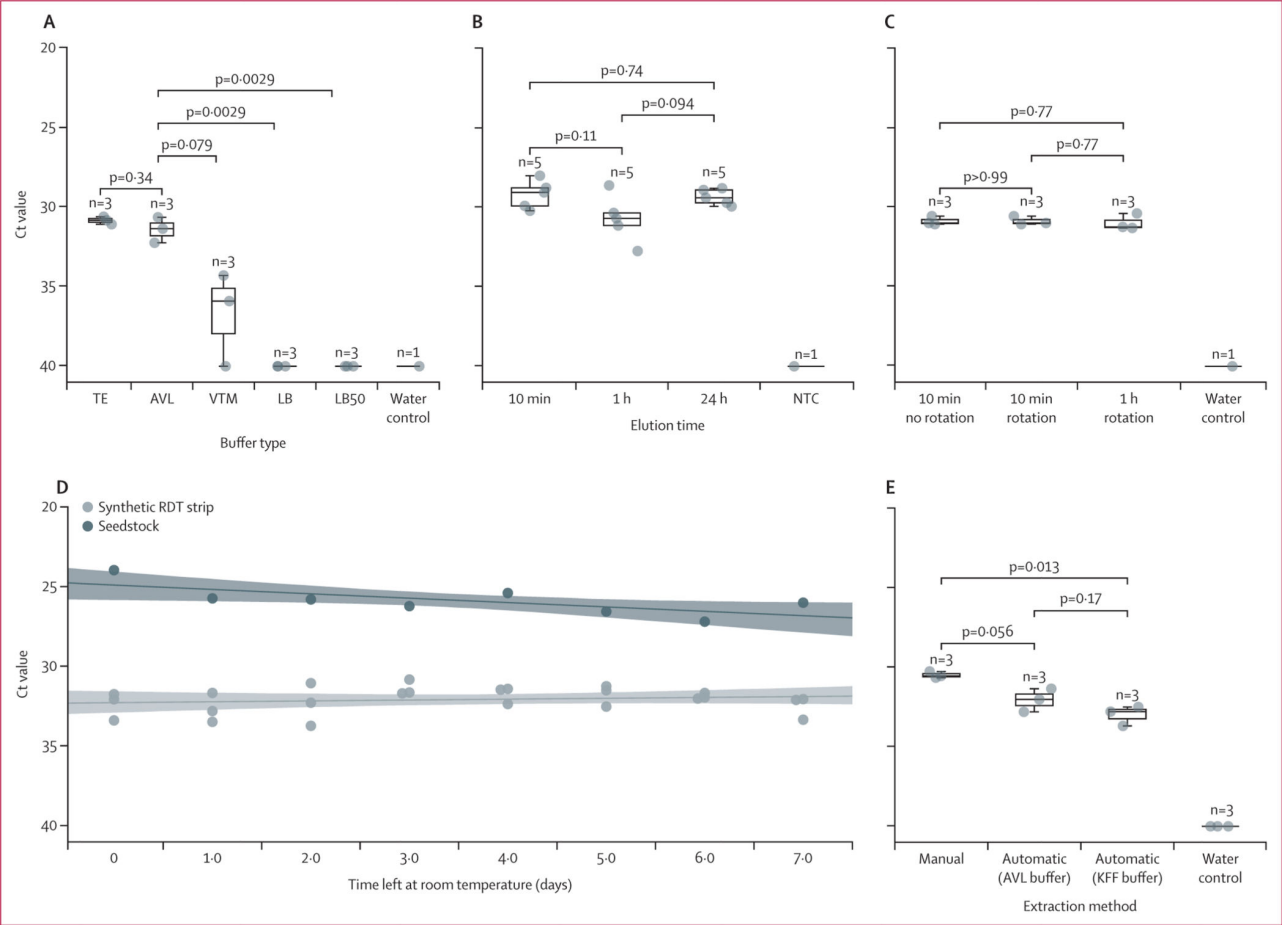
Optimisation of rapid diagnostic test elution via synthetic samples Data points are the medians of three technical PCR replicates. Boxplots show quartiles of the dataset, with whiskers extending to points within 1⋅5 IQR of the lower or upper quartile (all plotted with the seaborn.boxplot function). p values were calculated via *t* test. Water controls are negative controls that underwent RNA extraction, and NTCs were added to PCR post-extraction. (A) The Ct values (y axis; inverted) for synthetic samples that were eluted in five different buffers (x axis). (B–C) The Ct values (y axis; inverted) for synthetic samples that were eluted for varying time lengths (x axis). (C) Elution occurred with or without rotation (x axis). (D) The Ct values (y axis; inverted) for RDT strips (light grey) or viral seedstock (dark grey), plotted against the number of days that seedstock and seeded strips were left at room temperature. Least-squares linear regression models were fit using each set of data points; shaded areas represent 95% CIs. For strips, the slope was −0⋅06 (95% CI −0⋅23 to 0⋅10; y axis inverted). For seedstock, the slope was 0⋅33 (0⋅07 to 0⋅58; y axis inverted). (E) The Ct values (y axis; inverted) for synthetic samples that were extracted using either QIAamp spin-column extraction or KingFisher automated extraction. AVL=Buffer AVL. Ct=cycle threshold. KFF=KingFisher Flex System. LB=lysis buffer solution. NTC=no-template controls. RDT=rapid diagnostic test. TE=Tris EDTA buffer pH 8. VTM=Universal Viral Transport Medium.

We first optimised an extraction protocol to elute genetic material from the lateral flow strip of RDTs. We assessed the effect of using different buffers, and increased time or rotation during incubation, on elution efficacy of QuickVue strips. We found that viral RNA elution, measured by Ct, was greatest when using either AVL or TE, and was significantly poorer with LB or LB50 (figure 2A). We proceeded with AVL due to its additional use as a viral inactivation agent.^22^ We found that neither increasing the length of nor adding rotation to strip incubation affected elution efficacy (figure 2B, C). Finally, we found that viral RNA was stable on QuickVue strips for at least 1 week at room temperature (figure 2D). We moved forward with a protocol consisting of a 10 min benchtop incubation in buffer AVL, for QuickVue strips left at room temperature for a maximum of 1 week after testing. We next compared the compatibility of our elution protocol with manual (ie, column-based) and automated extraction. The manual method was more effective than automated extraction (relative loss of 2⋅0 Cts; figure 2E). We used manual extraction for further evaluation.

**Figure 3:**
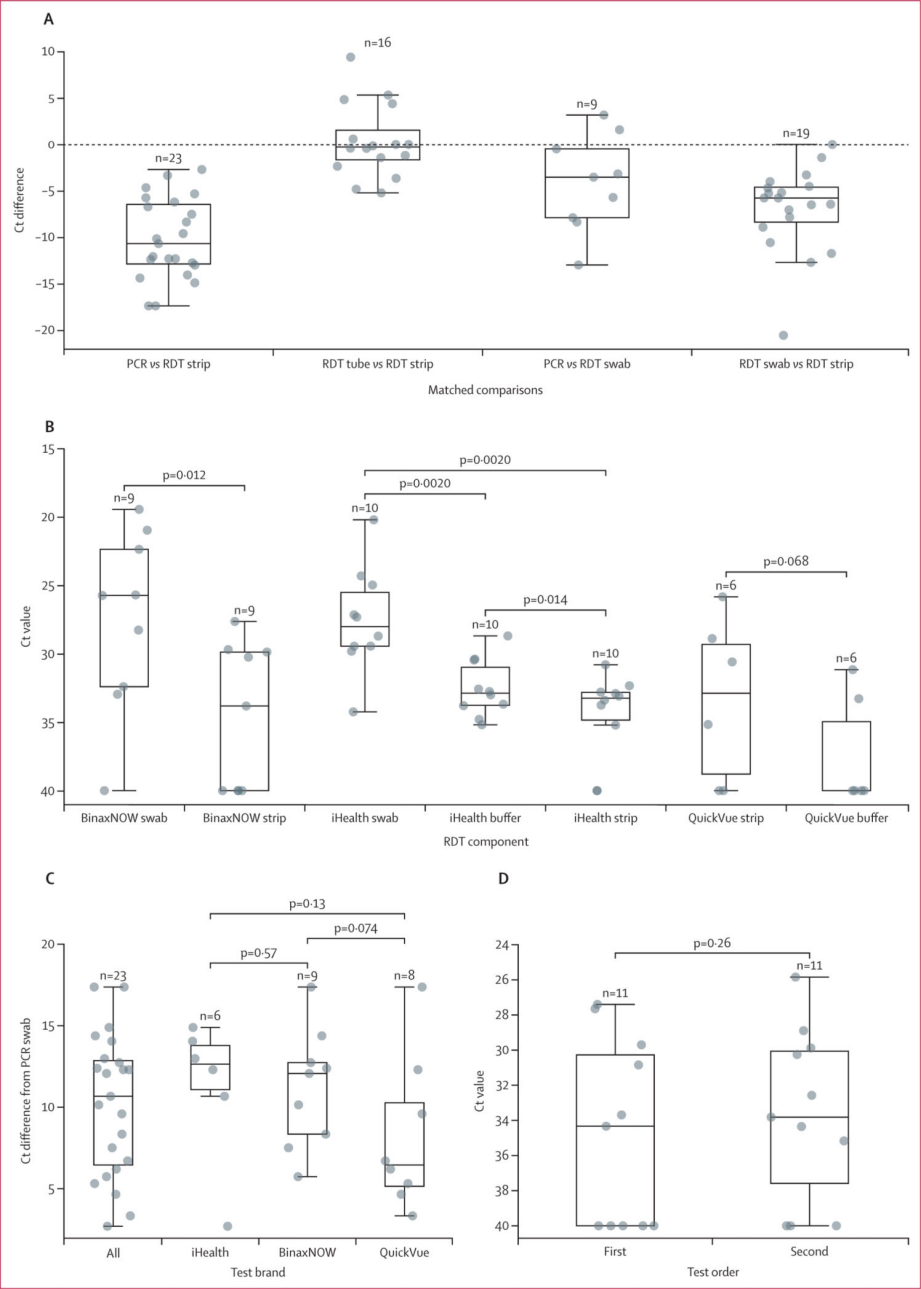
Evaluation of rapid diagnostic test extraction performance Plotted values are the medians of three technical PCR replicates. Boxplots show quartiles of the dataset, with whiskers extending to points within 1⋅5 IQR of the lower or upper quartile (all plotted with the seaborn.boxplot function). p values were calculated via Wilcoxon signed-rank test. (A) The difference in Ct values (y axis) between corresponding RDT components (swab, strip, and residual buffer) and polypropylene PCR swabs. The difference is calculated as the Ct value of the second member of the comparison subtracted from the Ct value of the first member of the comparison (ie, PCR *vs* RDT swab is calculated as each relevant individual’s RDT swab Ct value subtracted from their PCR Ct value). This analysis includes individuals from both cohorts. (B) The Ct values (y axis; inverted) for RDT components collected from SARS-CoV-2 positive individuals. This analysis includes individuals from either cohort who collected and returned all components of a particular RDT. (C) The difference in Ct values (y axis) between RDT strips (by brand) and corresponding polypropylene PCR swabs from SARS-CoV-2-positive individuals in cohort 1. (D) The Ct values (y axis; inverted) from the first and second RDT strips collected from SARS-CoV-2 positive individuals in cohort 1. Ct=cycle threshold. RDT=rapid diagnostic test.

We quantified the performance of our optimised extraction protocol on 38 RDT-derived samples and 13 PCR swabs collected from 13 individuals in cohort 1 (gender and age distributions are in appendix 1 p 2; no other demographic characteristics were collected), and 30 RDT-derived samples collected from ten individuals in cohort 2 (appendix 1 pp 5–8). We successfully amplified SARS-CoV-2 RNA from RDTs across brands and components, with differences in performance across components (table; figure 3A). Across brands, RDT strips performed significantly poorer than matched PCR swabs (mean Ct difference 10⋅2 Ct [SD 4⋅3], p<0⋅0001), and equivalently to matched RDT residual buffer (mean Ct difference 0⋅3 Ct [3⋅9], p=0⋅92). RDT swabs displayed better performance than RDT strips (mean Ct difference 6⋅9 [4⋅6], p=0⋅0002), and performed similarly to PCR swabs (mean Ct difference 4⋅1 [5⋅2], p=0⋅055). These comparisons (both among RDT components and between RDTs and PCR swabs) held true when subsetting by brand, with one exception: iHealth residual buffer amplified better than corresponding strips (mean Ct difference 1⋅9 [1⋅9], p=0⋅014; figure 3B). No brand of RDT strips significantly outperformed another (figure 3C). For individuals who took multiple RDTs, we found that the order of test administration did not affect viral titre (figure 3D).

**Figure 4:**
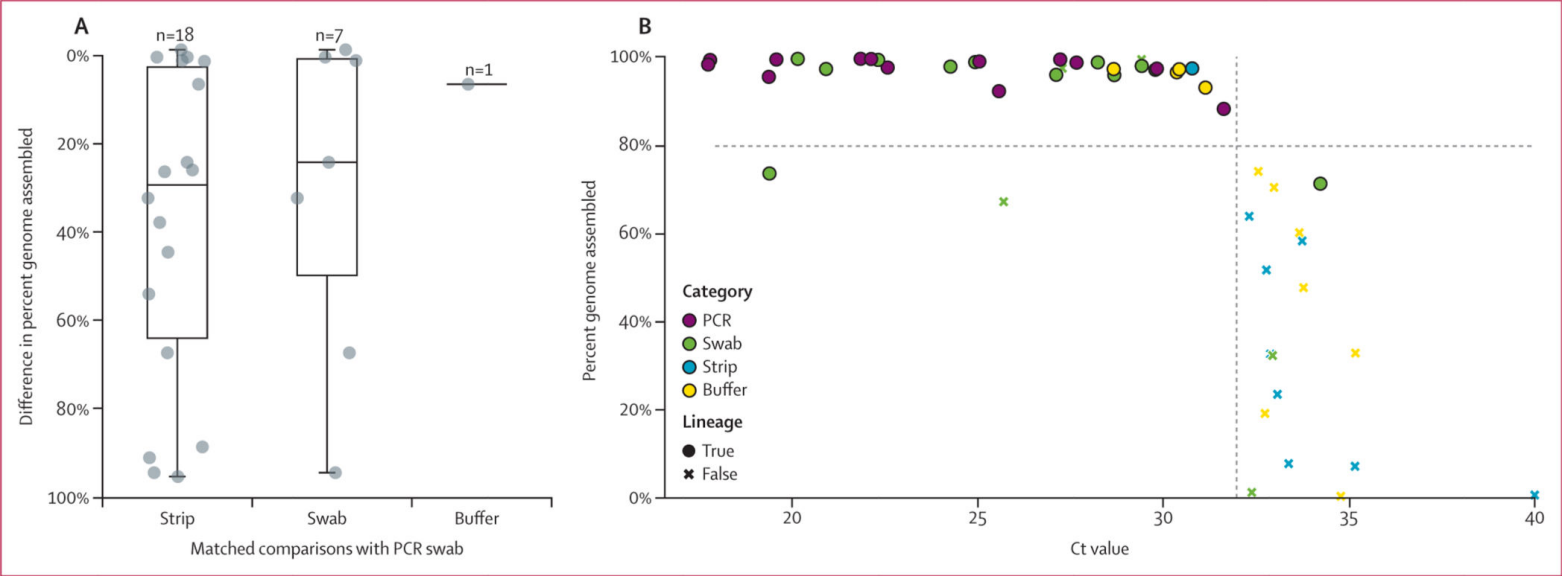
Evaluation of RDT sequencing performance (A) Difference in percent of unambiguous genome assembled from sequencing of RDT components and corresponding PCR swabs. (B) Percent of unambiguous genome assembled versus Ct value for genomes produced from RDT components or PCR swabs. Plotted values are the medians of three technical PCR replicates. Values are shown as circles or crosses to designate whether viral lineage assignment was possible. Dashed lines indicate a Ct value of 32 and a percent genome assembly of 80%. Ct=cycle threshold. RDT=rapid diagnostic test.

We next analysed the sequencing performance of 18 RDT-derived samples from cohort 1 and 28 samples from cohort 2 (appendix 1 pp 5–8). We assembled complete genomes from all 13 PCR swabs and 12 (71%) of 17 RDT swabs. However, we assembled complete genomes from only two (40%) of five QuickVue strips, one (9%) of 11 iHealth strips, and none of the BinaxNOW strips (table). Because even incomplete genomes can yield useful phylogenetic information, we compared the unambiguous genome length across RDT components. Although RDT strips performed significantly poorer than matched PCR swabs (mean 51% *vs* 97%; p<0⋅0001), RDT swabs performed comparably to PCR swabs (mean 67% *vs* 98%, p=0⋅078; figure 4A). Read depth strongly correlated with percent genome assembled (Spearman rank-order correlation coefficient of 0⋅915, p<0⋅0001; table). As expected, a significant fraction of the variability in sequencing length across all samples could be attributed to viral load (Spearman rank-order correlation coefficient of −0⋅80, p<0⋅0001). No samples with Ct greater than 32 assembled a complete (greater than 80%) viral genome (figure 4B).

A major use case of viral sequencing is viral lineage identification; we thus assessed our ability to assign lineages to RDT-derived samples. SARS-CoV-2 Pango lineage assignment was possible for all 13 PCR swabs, 14 (82%) of 17 RDT swabs, four (22%) of 18 RDT strips, and five (45%) of 11 RDT buffer samples (table). Lineages identified from samples from the same individual were consistent (appendix 1 p 7).

We assessed the fidelity of mutation calls produced from RDTs relative to matched PCR swabs and found high concordance among consensus-level SNVs. Only four mismatches were identified across more than 300 000 sites compared in pairwise consensus genomes. However, intrahost SNVs (iSNVs) showed little concordance (0–7%; appendix 1 p 14).

To examine the utility of RDT-derived sequences in a public health setting, we sequenced samples from a suspected outbreak of COVID-19 at a senior housing building in MA, USA. Ten positive RDT samples were successfully sequenced via amplicon-based sequencing. Negative RDT swabs from five symptomatic individuals were sequenced via metagenomics, producing two SARS-CoV-2 genomes from previously positive individuals.

**Figure 5:**
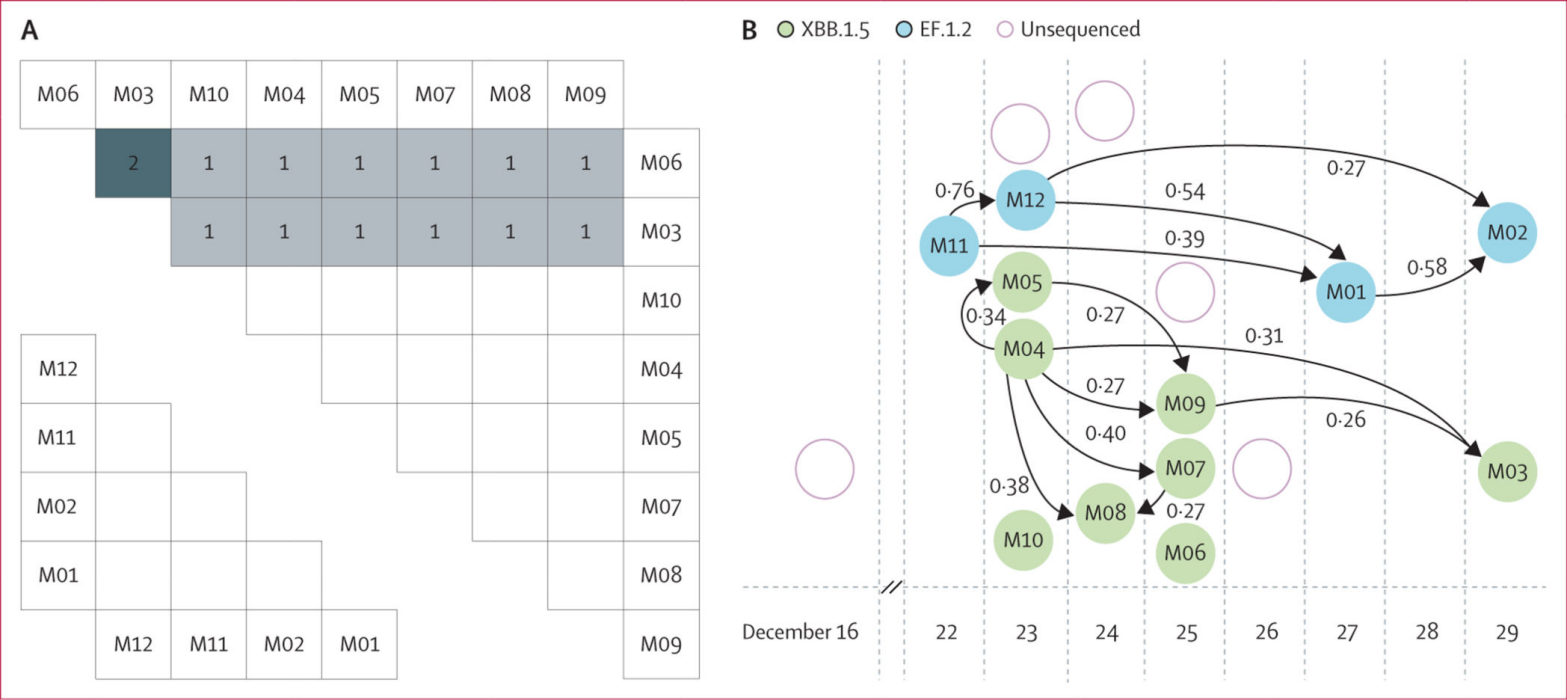
Identification of transmission links in a cluster (A) Pairwise SNV distance matrix for eight identified XBB.1.5 cases (top right) and four identified EF.1.2 cases (bottom left) in the outbreak. Number of SNV differences between each pair is shown in cells and is excluded for pairs that are identical (ie, SNV distance of 0). (B) Outbreak transmission reconstruction network, generated via outbreaker2, showing putative person-to-person transmission events. Circles represent SARS-CoV-2 positive individuals, circle colour indicates genome lineage, and arrows connecting circles indicate the presence and directionality of a putative transmission event. Arrows are annotated with the posterior probability of a given transmission and are shown only for transmission events with a posterior probability of at least 0⋅25. SNV=single-nucleotide variant.

The outbreak, which had a building-wide attack rate of at least 10⋅4% (17 of 164), consisted of at least two introductions: one of EF.1.2 (n=4; M1–M2 and M11–M12) and at least one of XBB.1.5 (n=8, M3–M10; appendix 1 p 3). The four EF.1.2 sequences were identical at the consensus level, and the XBB.1.5 cases contained zero to two pairwise mutations (figure 5A). Per our outbreak reconstruction, transmission links were identified for ten of the 12 individuals; M06 and M10 could not be confidently linked to specific other individuals within the building (figure 5B; appendix 1 p 4).

## Discussion

In this study, we optimised a protocol for elution of genetic material from RDT strips, consisting of a 10 min room temperature incubation in AVL for RDTs taken up to a week before processing. We demonstrated that RDT strips’ viral titres and genome lengths were significantly lower than those of PCR and RDT swabs, affecting viral lineage assignment. We found that RDT swabs performed better than other test components and similarly to gold-standard PCR swabs. Although RDTs yielded consensus-level mutation calls that paralleled those of PCR swabs, iSNVs in RDT samples were not replicated in matched PCR swabs; this finding suggests that only consensus-level mutations derived from RDT sequencing are trustworthy. Finally, although initial epidemiological investigations pointed towards a large social gathering as the event spurring transmissions from one index case in the senior living community outbreak, a genomic characterisation instead supports spread of multiple variants through social networks.

Although previous studies have introduced the potential of sequencing from RDTs,^5,6^ our study is unique in that it optimises the elution protocol, compares results across multiple RDT brands and components, uses person-matched PCR swabs, and establishes the utility of RDT sequencing in a real-time outbreak investigation with public health partners. Our findings also echo results seen in parallel fields. An overabundance of non-replicable iSNVs was previously noted for wastewater sequencing^4^ and for low titre PCR samples,^23^ and emphasises the need for technical replicates for high-fidelity iSNV calling.^24^ As shown previously for PCR swabs, Ct was a strong indicator of sequencing quality;^25^ we recommend implementing a Ct threshold of 32 when selecting RDT samples to sequence.

Cohort 1 allowed for an in-depth comparison of RDT brands and components. Although sequencing from residual PCR material is preferable to sequencing of RDT strips, we have shown that sequencing of RDT-derived swabs performs comparably to sequencing of PCR swabs. Through our optimisation of viral elution from RDT strips, we not only set best practices, but also introduce flexibility into the protocol. For example, we note the equivalency of AVL and TE buffers, the negligibility of additional incubation time, and the potential to scale via an automated extraction method. Finally, we used RDT sequencing to sequence samples in a timely manner for an outbreak of public health concern occurring during the holiday season. The nature of the local RDT-based testing drive enabled immediate diagnosis and isolation of positive individuals followed by same-day delivery of positive samples to our laboratory. The genomic sequencing lent support to a theory of multiple introductions, some with onward transmission.

Although our study lays the groundwork for sequencing of RDTs, we note that our study has some limitations. Although we used multiple brands of RDTs employing lateral flow technology, we did not test our protocol on all of the over 200 FDA-EUA-approved SARS-CoV-2 RDTs. Moreover, although we show that synthetic samples are stable at room temperature for up to a week, and that clinical RDTs extracted within 5 days of testing yielded genomes, it is possible that clinical samples degrade over time. To comprehensively assess this possibility would require individuals to take multiple RDTs on the same day for researchers to extract at different timepoints. Further, it is possible that temperature fluctuations during shipment could affect RDT stability; this hypothesis was not tested in our study. Finally, our study was conducted when omicron and its subvariants predominated, and it ispossible, althoughunlikely, that RDT sequencing of other viral lineages would show improved or reduced efficacy.

In summary, we have shown the efficacy of sequencing from RDT components and established current best practices for real-world use of this method. We anticipate that reliance upon RDTs will increase, making this protocol particularly important for the continued surveillance of SARS-CoV-2. When planning to sequence from RDTs, we recommend collection of the RDT swabs; when this is not possible, strips or residual buffers can also yield accurate, albeit fewer, genomic data. This protocol might be modifiable to allow for sequencing of other viruses, such as influenza or respiratory syncytial virus, from similar lateral-flow-based RDTs.

## Supplementary Material

1

2

## Figures and Tables

**Table:** Sequencing metrics of positive samples

	Extraction analyses			Sequencing analyses						
		
	Number of samples analysed	Number of RT-PCR positive samples (percent)	Mean (range; SD) of Ct values for positive samples	Number of samples analysed	Mean (SD) percent genome assembled	Number of samples yielding partial (>10%) genomes	Number of samples yielding complete (>80%) genomes	Mean (SD) read depth	Number of samples with assigned clade	Number of samples with assigned lineage

**Cohort 1 (n=13)**										
PCR swab	13	13 (100%)	23·7 (17·8–31·7; 4·5)	13	97% (3)	13	13	18 463 (14 317)	13	13
BinaxNOW strip	9	5 (56%)	30·3 (27·6–33·8; 2·2)	2	23% (32)	1	0	44 259 (62 592)	1	0
BinaxNOW swab	9	8 (89%)	26·0 (19·5–33·0; 5·0)	7	67% (38)	6	3	18 258 (15 076)	6	4
QuickVue strip	8	6 (75%)	29·6 (25·8–35·2; 3·2)	5	66% (38)	4	2	21 250 (16 975)	4	3
QuickVue buffer	6	2 (33%)	32·2 (31·2–33·3; 1·5)	1	93%	1	1	5007	1	1
iHealth strip	6	5 (83%)	33·2 (30·8–34·3; 1·5)	3	44% (32)	2	0	7487 (7658)	2	0
**Cohort 2 (n=10)**										
iHealth strip	10	8 (80%)	33·0 (30·8–35·2; 1·2)	8	43% (31)	6	1	5677 (8489)	6	1
iHealth swab	10	10 (100%)	27·6 (20·2–34·2; 3·8)	10	95% (9)	10	9	17 902 (15 637)	10	10
iHealth buffer	10	10 (100%)	32·5 (28·7–35·2; 2·1)	10	59% (34)	9	3	10 437 (12 175)	8	4

Sequencing metrics of positive samples, categorised by cohort, test type (ie, RDT brand or PCR swab), and component (ie, strip, swab, or tube). Ct=cycle threshold. RDT=rapid diagnostic test.

## Research in context

### Evidence before this study

We searched PubMed from database inception to April 27, 2023, for studies with search terms (SARS-CoV-2) AND (sequence OR sequencing) AND (rapid OR antigen) in the title or abstract. No language restrictions were applied to this search. The search produced 1192 hits, the majority of which were unrelated to genomic sequencing of rapid diagnostic tests (RDTs). We limited studies to primary research articles and preprints (ie, we removed reviews, editorials, and clinical trials). The search yielded three relevant studies, each of which provided proof-of-concept data on the ability to sequence SARS-CoV-2 from synthetic or clinical rapid tests. The studies used distinct sequencing methods without protocol optimisation and did not compare the efficacy of sequencing from RDTs relative to person-matched PCR tests. A Google Scholar search for “SARS-CoV-2 rapid antigen test sequencing” on April 27, 2023, with no language restrictions, revealed two additional studies: one also provided proof-of-concept of RDT sequencing capability for both SARS-CoV-2 and influenza, and the other demonstrated that RDT swabs with a cycle threshold value below 30 could yield SARS-CoV-2 sequences. In summary, it was clear that RDTs could yield SARS-CoV-2 genomes before this study.

### Added value of this study

To the best of our knowledge, in this study, we conduct the first optimisation of an elution protocol to maximise viral nucleic acid recovery from RDTs. Our study also offers the first rigorous assessment of RDT sequencing relative to the gold-standard approach (ie, sequencing residual material from PCR swabs). We evaluate both sequence yield (eg, assembled genome length) and fidelity (eg, accuracy of mutation calls and lineage designations). We conduct a rigorous examination of sequence quality across various RDT brands and components (ie, RDT swabs, lateral flow strips, and residual buffer). Finally, we demonstrate that RDT sequencing data can be used for outbreak investigation and transmission inference.

### Implications of all the available evidence

Taken together, previous studies and our findings show that viral genomes can be generated from RDTs. Moreover, we establish best practices for real-world implementation of RDT sequencing and show the fidelity of RDT-derived genomes for viral lineage assignment and transmission inference. However, our data suggest that RDT-derived intrahost variants, even when subject to stringent filtering, are not reliably recapitulated in person-matched PCR swabs. Nevertheless, we anticipate that reliance upon RDTs will increase, making this protocol particularly important for the continued surveillance of SARS-CoV-2. This study also serves as a template for the sequencing of other viruses, such as influenza or respiratory syncytial virus, from similar lateral-flow-based RDTs.
