# Supplementary material for: Optimisation and evaluation of viral genomic sequencing of SARS-CoV-2 rapid diagnostic tests: a laboratory and cohort-based study
Source: Lancet Microbe. Author manuscript; Available in PMC 2024 Aug 14. (PMC11322816; doi:10.1016/S2666-5247(23)00399-3)
Supplement: 1 [file NIHMS1992475-supplement-1.pdf]

THE LANCET
Microbe

Supplementary appendix 1

This appendix formed part of the original submission and has been peer reviewed.
We post it as supplied by the authors.

Supplement to: Paull JS, Petros BA, Brock-Fisher TM, et al. Optimisation and evaluation of viral genomic sequencing of SARS-CoV-2 rapid diagnostic tests: a laboratory and cohort-based study. *Lancet Microbe* 2024. https://doi.org/10.1016/S2666-5247(23)00399-3

Table of contents

Supplemental Figure 1 – Gender and age distribution among participants in cohort 1
pg 2

Supplemental Figure 2 – Phylogenetic tree of outbreak-associated viral sequences
pg 3

Supplemental Figure 3 – Distribution of transmission linkage probabilities
pg 4

Supplemental Figure 4 – Flowchart of sample inclusion and exclusion
Pg 5-6

Supplemental Table 1 – Breakdown of samples received and analyzed
pg 7-8

Supplemental Table 2 – Lineages assigned to each sample from each participant
pg 9

Supplemental Table 3 – NCBI accession numbers
pg 10-12

Supplemental Table 4 – Suppliers for all reagents, kits, and equipment
pg 13

Supplemental Table 5 – Concordance of SNVs and iSNVs between RDTs and PCR swabs
pg 14

Supplemental Table 6 – Number of viral reads in samples sequenced via metagenomics
pg 15

Supplemental Methods
pg 16-17

Supplemental Document 1 – Instructions sent to individuals in cohort 1
pg 18-23

Supplemental Document 2 – Questionnaire given verbally to individuals in cohort 2
pg 24-29

Supplemental Figure 1 – Gender and age distribution among participants in cohort 1

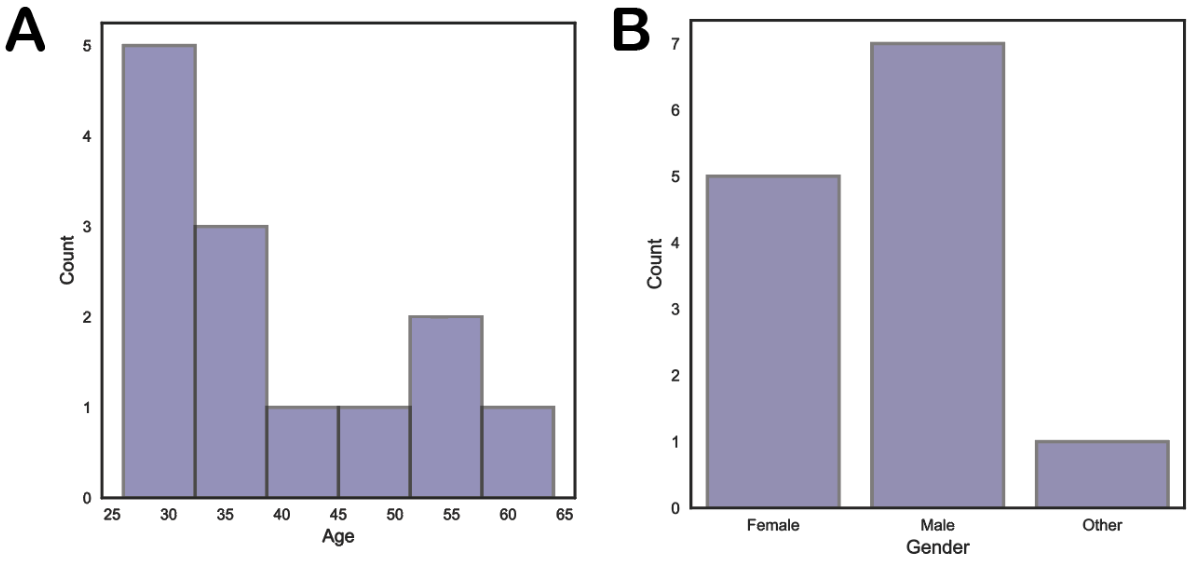

A-B. Distribution of age (**A**) and self-reported gender (**B**) among SARS-CoV-2 positive individuals in cohort 1.

Supplemental Figure 2 – Phylogenetic tree of outbreak-associated viral sequences

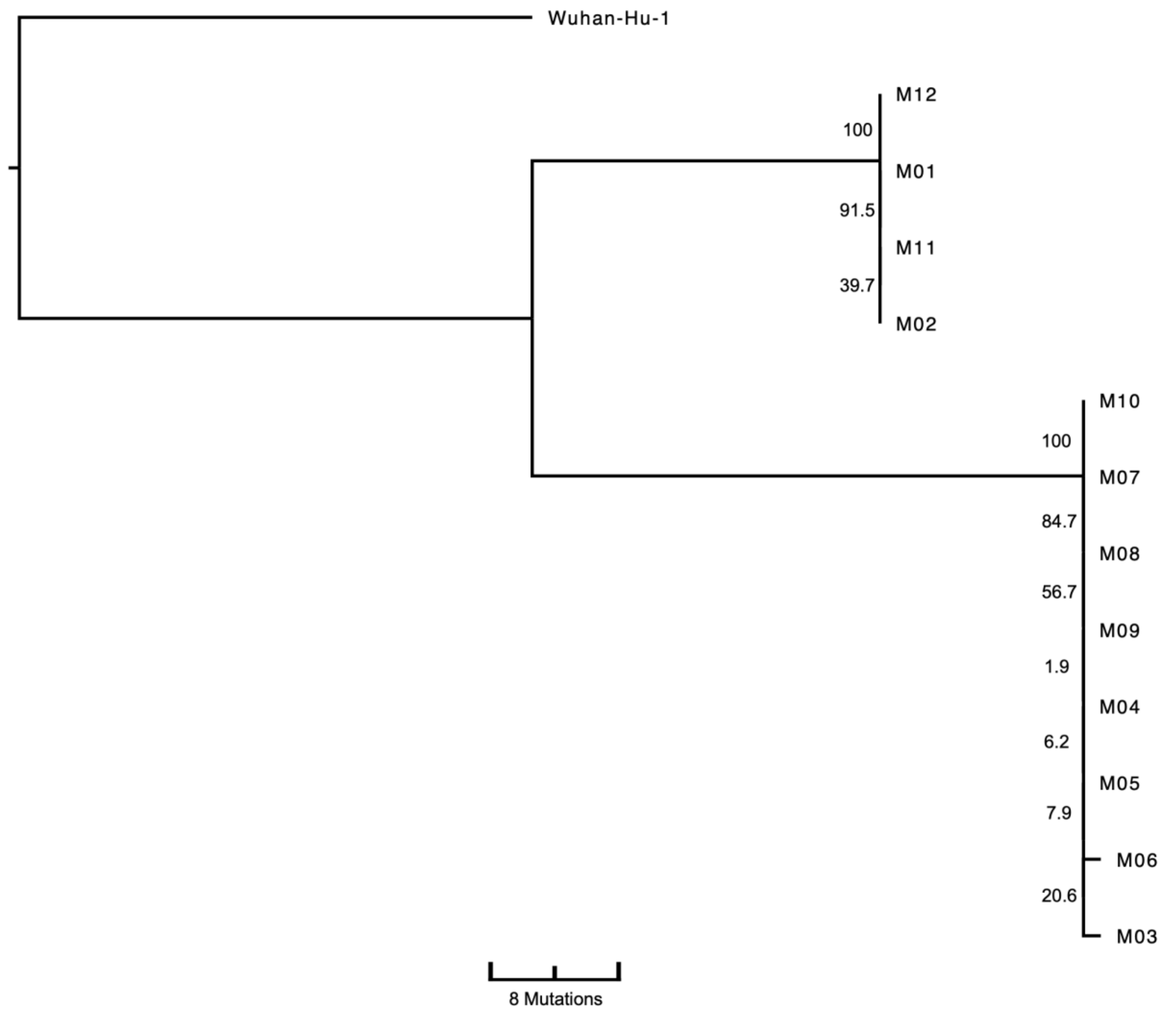

Maximum-likelihood phylogeny of viral genomes associated with the senior living facility outbreak, rooted on the Wuhan-Hu-1 sequence. The scale bar denotes substitutions. Bootstrap values are shown.

Supplemental Figure 3 – Distribution of transmission linkage probabilities

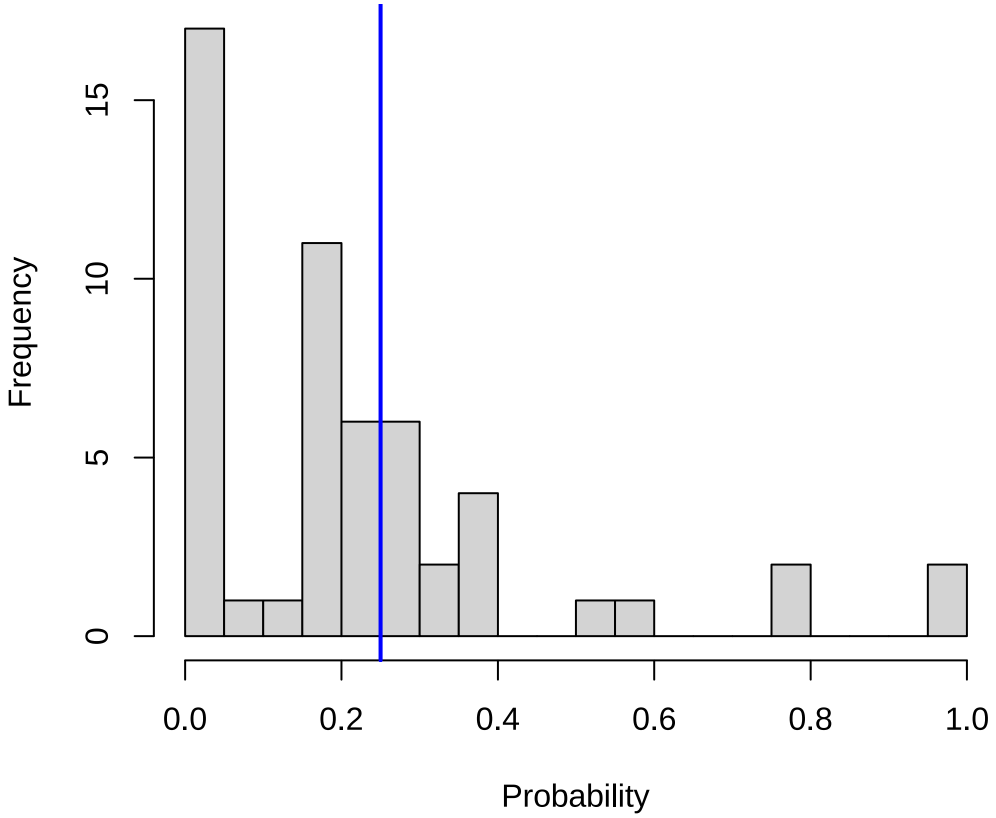

Distribution of pairwise transmission linkage probabilities resulting from outbreak reconstruction with outbreaker2. The blue line at x = 0.25 represents the threshold used to call putative transmission links in Figure 5B.

Supplemental Figure 4 – Flowchart of sample inclusion and exclusion

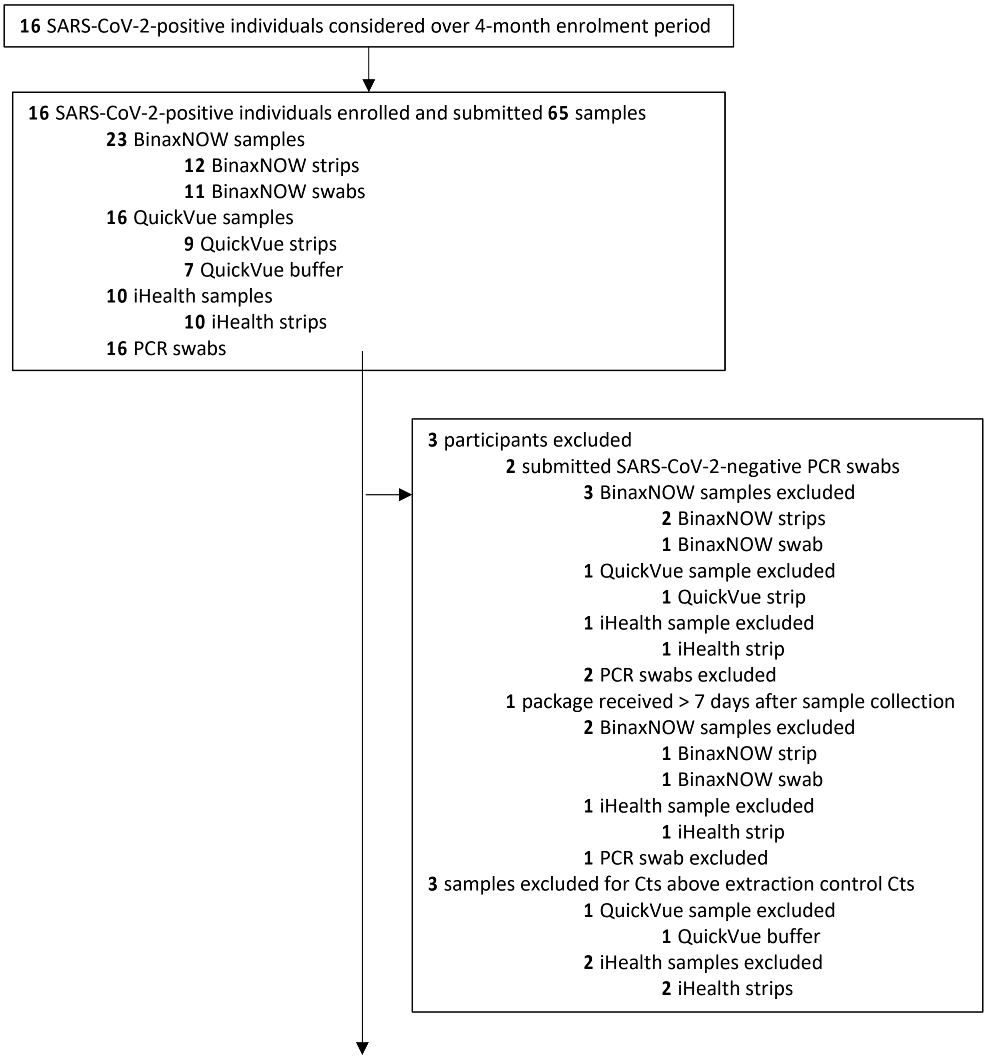

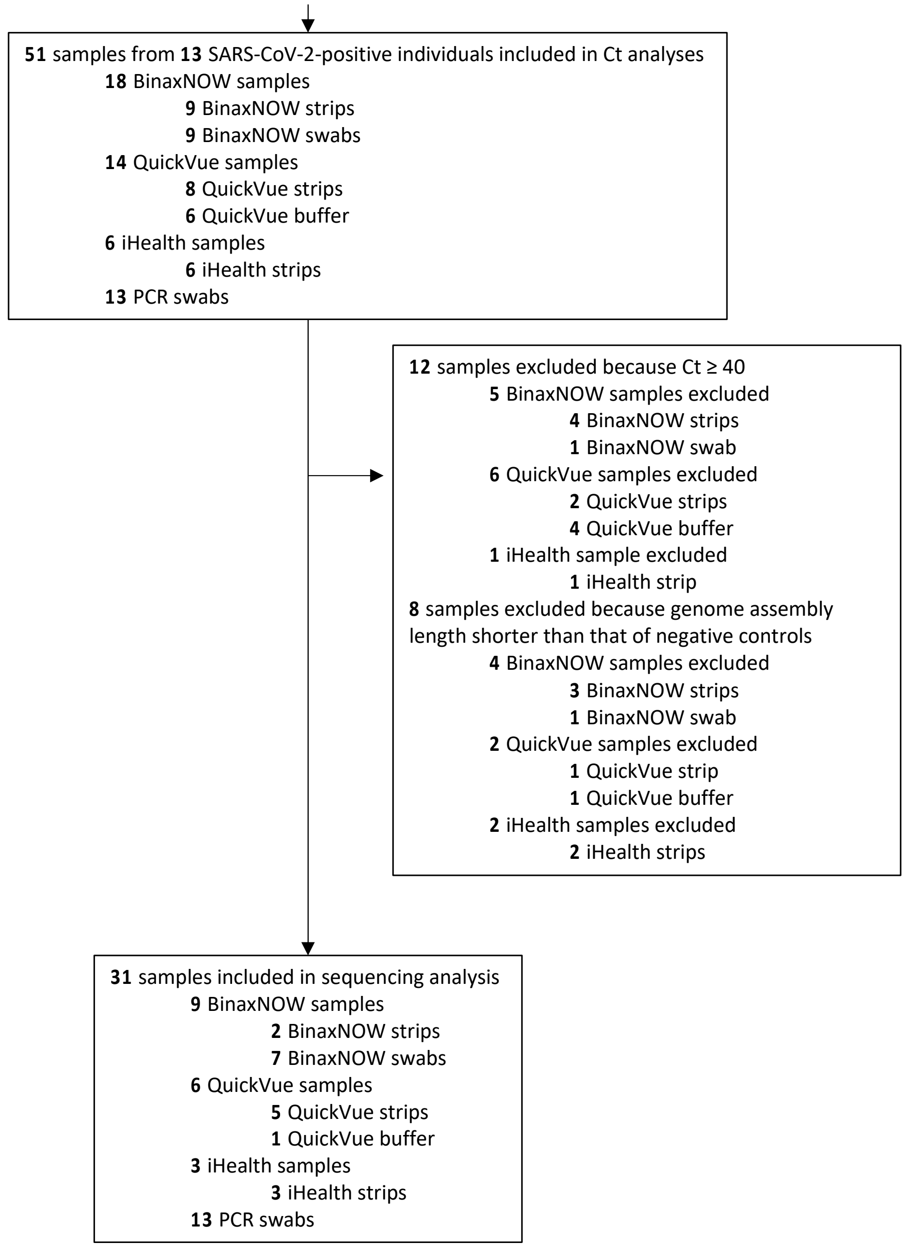

Graphical description of sample flow throughout the analyses, including reasons for sample inclusion or exclusion at different stages of analyses.

Supplemental Table 1 – Breakdown of RDT-derived samples received and analyzed

This table shows all RDT components that we received per enrolled individual. Samples are named per our external (column 1) and internal naming systems (column 2). B, Q, and iH represent BinaxNow, QuickVue, and iHealth. str/strip, swab, and tube/liquid represent strips, swabs, and residual buffer components. All RDTs from cohort 2 (bottom half) were iHealth. We show the number of sample components received (column 3) and detail samples that were excluded from analyses (columns 4-6).

**Individual (cohort 1)**	**RDTs sent to the lab**	**Number of RDT components received**	**Samples excluded from Ct analysis**	**Samples with no detected viral titer**	**Samples excluded from sequencing analysis**
1	01_B1_swab, 01_B2_str, 01_Q1_tube, 01_Q2_str	4		01_B2_str, 01_Q1_tube	
2	02_B2_str, 02_Q2_str	2	All samples; PCR swab was negative		
3	03_B1_swab, 03_B2_str, 03_iH	3	All samples; PCR swab was negative		
4	04_B1_str, 04_B2_swab, 04_iH	3			04_B1_str and 04_iH; genomes shorter than extraction control genomes
5	05_Q1_str, 05_Q2_tube, 05_iH	3		05_Q1_str, 05_Q2_tube, 05_iH	
6	06_iH, 06_Q1_str, 06_Q2_tube	3			06_Q1_str and 06_iH; genomes shorter than extraction control genomes
7	07_B1_str, 07_B2_swab, 07_Q1_str, 07_Q2_tube	4		07_B1_str, 07_Q2_tube	
8	08_B1_str, 08_B2_swab, 08_Q1_str, 08_Q2_tube	4		08_B1_str, 08_Q1_str, 08_Q2_tube	
9	09_iH, 09_B1_str, 09_B2_swab	3			
10	10_B1_str, 10_B2_swab, 10_iH	3			
11	11_iH	1	11_iH; Ct higher than extraction control Ct		
12	12_iH, 12_Q1_str	2	12_iH; Ct higher than extraction control Ct		
13	13_B1_str, 13_B2_swab, 13_Q1_str, 13_Q2_tube	4			13_B1_str, 13_B2_swab, 13_Q2_tube; genomes shorter than extraction control genomes
14	14_Q1_str, 14_Q2_tube, 14_B1_str, 14_B2_swab	4	14_Q2_tube; Ct higher than extraction control Ct		14_B1_str; genome shorter than extraction control genomes
15	15_iH, 15_B1_swab, 15_B2_str	3	All samples; received weeks after collection		
16	16_B1_swab, 16_B2_str, 16_iH	3		16_B1_swab, 16_B2_str	
	Cohort 1	**Number of RDTs received:**	**Number of RDT components included in Ct analysis:**	**Number of RDT components that underwent amplicon sequencing:**	**Number of RDT components included in sequencing analysis:**
	**Count of samples:**	**49**	**38**	**26**	**18**
**Individual (cohort 2)**	**RDTs sent to the lab**	**Number of RDT components received**	**Samples excluded from Ct analysis**	**Samples with no detected viral titer**	**Samples excluded from sequencing analysis**
M01	209_liquid, 209_strip, 209_swab	3			
M02	314_liquid, 314_strip, 314_swab	3		314_strip	
M03	301_liquid, 301_strip, 301_swab	3			
M04	315_liquid, 315_strip, 315_swab	3			
M05	407_liquid, 407_strip, 407_swab	3			

M06	501_liquid, 501_strip, 501_swab	3			
M07	514_liquid, 514_strip, 514_swab	3			
M08	518_liquid, 518_strip, 518_swab	3			
M09	808_liquid, 808_strip, 808_swab	3			
M10	904_liquid, 904_strip, 904_swab	3		904_strip	
M11	308_liquid, 308_strip, 308_swab	3	All samples excluded from analysis due to negative RDTs		Swab sample metagenome-assembled genomes included in outbreak analysis only
M12	215_liquid, 215_strip, 215_swab	3			
M13	203_liquid, 203_strip, 203_swab	3			Metagenomic sequencing of swab samples did not yield viral genomes
M14	716_liquid, 716_strip, 716_swab	3			
M15	913_liquid, 913_strip, 913_swab	3			
	Cohort 2	**Number of RDT components received:**	**Number of RDT components included in Ct analysis:**	**Number of RDT components that underwent amplicon sequencing and analysis:**	**Number of RDT components that underwent metagenomic analysis:**
	**Count of samples:**	**45**	**30**	**28**	**3**

Supplemental Table 2 – Lineages assigned to each sample from each participant

For each individual enrolled (first column) in either cohort 1 (C1) or cohort 2 (C2), we list the assigned lineage and clade of each sequenced sample.

	**PCR**	**QuickVue Strip**	**QuickVue Buffer**	**iHealth Strip**	**iHealth Buffer**	**iHealth Swab**	**Binax Strip**	**Binax Swab**
**C1-01**	22B BA.5.2.1	22B Unassigned						22B Unassigned
**C1-04**	22D BA.2.75.2							22D Unassigned
**C1-05**	22B BF.21							
**C1-06**	22B BF.27		22B BF.27					
**C1-07**	22B BA.5.2.1	Recombinant Unassigned						22B BA.5.2.1
**C1-08**	22A BA.4.6							22A BA.4.6
**C1-09**	22B BA.5.2			Recombinant Unassigned				Recombinant Unassigned
**C1-10**	22B BE.1.1			22B Unassigned			22B Unassigned	22B BE.1.1
**C1-11**	22B BE.1.1							
**C1-12**	22B BF.26	22B BF.26						
**C1-13**	22A BA.4.6	22A BA.4.6						
**C1-14**	22A BA.4.6	22A BA.4.6						22A BA.4.6
**C1-16**	22B BA.5.2			22B Unassigned				
**C2-M01**				22E Unassigned	22E EF.1.2	22E EF.1.2		
**C2-M02**						22E EF.1.2		
**C2-M03**				22F Unassigned	23A Unassigned	23A XBB.1.5		
**C2-M04**				23A XBB.1.5	23A XBB.1.5	23A XBB.1.5		
**C2-M05**				Recombinant Unassigned	22F Unassigned	23A XBB.1.5		
**C2-M06**				23A Unassigned	23A XBB.1.5	23A XBB.1.5		
**C2-M07**				22F Unassigned	23A XBB.1.5	23A XBB.1.5		
**C2-M08**				Recombinant Unassigned	23A Unassigned	23A XBB.1.5		
**C2-M09**				22F Unassigned	Recombinant Unassigned	23A XBB.1.5		
**C2-M10**					22F Unassigned	23A XBB.1.5		
**C2-M11**						22E EF.1.2		
**C2-M12**						22E EF.1.2		

Supplemental Table 3 – NCBI accession numbers

This table lists Sequence Read Archive (SRA), BioProject, and BioSample accession numbers for all samples submitted to the SRA. The highest quality genome from any given individual was submitted to GenBank. The first column provides individual identifiers: C1 denotes cohort 1 and C2 denotes cohort 2. The second column provides the internal lab identifier. B, Q, and iH represent BinaxNow, QuickVue, and iHealth. Str/strip, swab, and tube/liquid represent strips, swabs, and residual buffer components. All tests from cohort 2 (bottom half) were iHealth RDTs. The remaining columns contain accession numbers.

Individual ID	Library ID	SRA accession	GenBank accession	BioProject accession	BioSample accession
C1_01	01_B1_swab	SRR24737964		PRJNA971982	SAMN35364755
C1_01	01_Q2_strip	SRR24737963		PRJNA971982	SAMN35364756
C1_01	01_R	SRR24737913	OR083045	PRJNA971982	SAMN35061199
C1_04	04_B1_str	SRR24737952		PRJNA971982	SAMN35364757
C1_04	04_B2_swab	SRR24737941		PRJNA971982	SAMN35364758
C1_04	04_iH	SRR24737930		PRJNA971982	SAMN35364759
C1_04	04_R	SRR24737912	OR083046	PRJNA971982	SAMN35061200
C1_05	05_Q1_str	SRR24737919		PRJNA971982	SAMN35364760
C1_05	05_R	SRR24737901	OR083047	PRJNA971982	SAMN35061201
C1_06	06_iH	SRR24737916		PRJNA971982	SAMN35364762
C1_06	06_Q1_str	SRR24737917		PRJNA971982	SAMN35364761
C1_06	06_Q2_tube	SRR24737890		PRJNA971982	SAMN35061202
C1_06	06_R	SRR24737887	OR083048	PRJNA971982	SAMN35061203
C1_07	07_B2_swab	SRR24737886		PRJNA971982	SAMN35061204
C1_07	07_Q1_str	SRR24737915		PRJNA971982	SAMN35364763
C1_07	07_R	SRR24737885	OR083049	PRJNA971982	SAMN35061205
C1_08	08_B2_swab	SRR24737914		PRJNA971982	SAMN35364764
C1_08	08_Q1_str	SRR24737962		PRJNA971982	SAMN35364765
C1_08	08_Q2_tube	SRR24737961		PRJNA971982	SAMN35364766
C1_08	08_R	SRR24737884	OR083050	PRJNA971982	SAMN35061206
C1_09	09_B1_str	SRR24737960		PRJNA971982	SAMN35364767
C1_09	09_B2_swab	SRR24737959		PRJNA971982	SAMN35364768
C1_09	09_iH	SRR24737958		PRJNA971982	SAMN35364769
C1_09	09_R	SRR24737883	OR083051	PRJNA971982	SAMN35061207
C1_10	10_B1_str	SRR24737957		PRJNA971982	SAMN35364770
C1_10	10_B2_swab	SRR24737882		PRJNA971982	SAMN35061208
C1_10	10_iH	SRR24737956		PRJNA971982	SAMN35364771
C1_10	10_R	SRR24737911	OR083052	PRJNA971982	SAMN35061209
C1_11	11_iH	SRR24737955		PRJNA971982	SAMN35364772
C1_11	11_R	SRR24737910	OR083053	PRJNA971982	SAMN35061210
C1_12	12_iH	SRR24737954		PRJNA971982	SAMN35364773
C1_12	12_Q1_str	SRR24737909		PRJNA971982	SAMN35061211

C1_12	12_R	SRR24737908	OR083054	PRJNA971982	SAMN35061212
C1_13	13_B1_str	SRR24737953		PRJNA971982	SAMN35364774
C1_13	13_B2_swab	SRR24737951		PRJNA971982	SAMN35364775
C1_13	13_Q1_str	SRR24737907		PRJNA971982	SAMN35061213
C1_13	13_Q2_tube	SRR24737950		PRJNA971982	SAMN35364776
C1_13	13_R	SRR24737906	OR083055	PRJNA971982	SAMN35061214
C1_14	14_B1_str	SRR24737949		PRJNA971982	SAMN35364777
C1_14	14_B2_swab	SRR24737905		PRJNA971982	SAMN35061215
C1_14	14_Q1_str	SRR24737948		PRJNA971982	SAMN35364778
C1_14	14_Q2_tube	SRR24737947		PRJNA971982	SAMN35364779
C1_14	14_R	SRR24737904	OR083056	PRJNA971982	SAMN35061216
C1_16	16_B1_swab	SRR24737946		PRJNA971982	SAMN35364780
C1_16	16_B2_str	SRR24737945		PRJNA971982	SAMN35364781
C1_16	16_iH	SRR24737944		PRJNA971982	SAMN35364782
C1_16	16_R	SRR24737903	OR083057	PRJNA971982	SAMN35061217
C2_M01	209_liquid	SRR24737943		PRJNA971982	SAMN35364783
C2_M01	209_strip	SRR24737942		PRJNA971982	SAMN35364784
C2_M01	209_swab	SRR24737902	OR083058	PRJNA971982	SAMN35061218
C2_M02	314_liquid	SRR24737938		PRJNA971982	SAMN35364787
C2_M02	314_strip	SRR24737937		PRJNA971982	SAMN35364788
C2_M02	314_swab	SRR24737899	OR083060	PRJNA971982	SAMN35061220
C2_M03	301_liquid	SRR24737940		PRJNA971982	SAMN35364785
C2_M03	301_strip	SRR24737939		PRJNA971982	SAMN35364786
C2_M03	301_swab	SRR24737900	OR083059	PRJNA971982	SAMN35061219
C2_M04	315_liquid	SRR24737898		PRJNA971982	SAMN35061221
C2_M04	315_strip	SRR24737897		PRJNA971982	SAMN35061222
C2_M04	315_swab	SRR24737896	OR083061	PRJNA971982	SAMN35061223
C2_M05	407_liquid	SRR24737936		PRJNA971982	SAMN35364789
C2_M05	407_strip	SRR24737935		PRJNA971982	SAMN35364790
C2_M05	407_swab	SRR24737895	OR083062	PRJNA971982	SAMN35061224
C2_M06	501_liquid	SRR24737894		PRJNA971982	SAMN35061225
C2_M06	501_strip	SRR24737931		PRJNA971982	SAMN35364794
C2_M06	501_swab	SRR24737893	OR083063	PRJNA971982	SAMN35061226
C2_M07	514_liquid	SRR24737892		PRJNA971982	SAMN35061227
C2_M07	514_strip	SRR24737926		PRJNA971982	SAMN35364798
C2_M07	514_swab	SRR24737891	OR083064	PRJNA971982	SAMN35061228
C2_M08	518_liquid	SRR24737925		PRJNA971982	SAMN35364799
C2_M08	518_strip	SRR24737924		PRJNA971982	SAMN35364800
C2_M08	518_swab	SRR24737889	OR083065	PRJNA971982	SAMN35061229

C2_M09	808_liquid	SRR24737923		PRJNA971982	SAMN35364801
C2_M09	808_strip	SRR24737922		PRJNA971982	SAMN35364802
C2_M09	808_swab	SRR24737888	OR083066	PRJNA971982	SAMN35061230
C2_M10	904_liquid	SRR24737921		PRJNA971982	SAMN35364803
C2_M10	904_strip	SRR24737920		PRJNA971982	SAMN35364804
C2_M10	904_swab	SRR24737918		PRJNA971982	SAMN35364805
C2_M11	308_swab	SRR26217800	OR614029	PRJNA971982	SAMN37605738
C2_M12	215_swab	SRR26217799	OR614028	PRJNA971982	SAMN37605739

Supplemental Table 4 – Suppliers for all reagents, kits, and equipment

Here we list all reagents, kits, and equipment referenced in the Methods section, alongside the supplier, supplier location, and product number or identifier.

Name of reagent, kit, equipment	Supplier	Location	Identifier
BinaxNow RDT	Abbott	150 S. Saunders Rd, Lake Forest, IL 60045, USA	
QuickVue At-Home OTC COVID-19 Test	Quidel	San Diego, California, USA	
iHealth	iHealth labs	San Jose, California, USA	
RHINOstic® Automated Swab for anterior nasal sampling	RHINOstics	Waltham, Massachusetts, USA	#RH-S000001
Heat-inactivated SARS-CoV-2	ATCC	Manassas, Virginia, USA	#VR-1986HK
Buffer AVL	Qiagen	Germantown, Maryland, USA	#19073
Lysis Buffer Solution	Sigma-Aldirch	St. Louis, Missouri, USA	#L1912
Universal Viral Transport Medium	BD	Franklin Lakes, New Jersey, USA	#220244
Tris EDTA Buffer pH 8	Thermo Fisher Scientific	Waltham, MA, USA	#J75793.AE
Thermo Scientific Digital Tube Revolver	Thermo Fisher Scientific	Waltham, MA, USA	#T9FB2186180
QIAamp Viral RNA Mini Kit	Qiagen	Germantown, Maryland, USA	#52904
MagMAX mirVana Total RNA Isolation Kit	Thermo Fisher Scientific	Waltham, MA, USA	#A27828
KingFisher Flex System	Thermo Fisher Scientific	Waltham, MA, USA	#5400630
TURBO DNase	Thermo Fisher Scientific	Waltham, MA, USA	#AM2238
RNAClean XP	Beckman Coulter	Brea, California, USA	#A66514
SARS-CoV-2 Research Use Only qPCR Primer & Probe Kit	Integrated DNA Technologies	Coralville, Iowa, USA	#10006713
TaqPath 1-Step RT-qPCR Master Mix	Thermo Fisher Scientific	Waltham, MA, USA	#A15300
QuantStudio 6 Flex Real-Time PCR System	Thermo Fisher Scientific	Waltham, MA, USA	#4485691
Illumina DNA Prep	Illumina	San Diego, California, USA	#20060059
ARTIC v4.1 primer set	Integrated DNA Technologies	Coralville, Iowa, USA	#10011442
NextSeq 550	Illumina	San Diego, California, USA	
Random hexamers	Thermo Fisher Scientific	Waltham, MA, USA	#N8080127
Nextera XT Kit	Illumina	San Diego, California, USA	#FC-131-1096
Twist Bioscience Respiratory Viral Research Panel	Twist Bioscience	San Francisco, California, USA	#103550
MiSeq Reagent Kit v2	Illumina	San Diego, California, USA	#MS-102-2002
MiSeq	Illumina	San Diego, California, USA	
SSIV RT master mix	Thermo Fisher Scientific	Waltham, MA, USA	#18090050
Random primers	Thermo Fisher Scientific	Waltham, MA, USA	#48190011
dNTPs	New England biolabs	Ipswich, MA, USA	#N0447S
SUPERase-In RNase inhibitor	Thermo Fisher Scientific	Waltham, MA, USA	#AM2696
RNase H	New England biolabs	Ipswich, MA, USA	#M0297S
Q5 Hot Start High-Fidelity 2X Master Mix	New England biolabs	Ipswich, MA, USA	#M0494
QUBIT dsDNA High Sensitivity Assay Kit	Thermo Fisher Scientific	Waltham, MA, USA	#Q32854
High Sensitivity D1000 Reagents	Agilent	Lexington, MA, USA	#5067-5585
High Sensitivity D1000 ScreenTape	Agilent	Lexington, MA, USA	#5067-5582
dNTPs	New England Biolabs	Ipswich, MA, USA	N0447S
SuperScript IV kit	Life Technologies	Carlsbad, CA, USA	#18090-010
NEBNext Second Strand Synthesis	New England Biolabs	Ipswich, MA, USA	B6117S
DNA Polymerase I, E. coli	New England Biolabs	Ipswich, MA, USA	M0209L
DNA Ligase, E. coli	New England Biolabs	Ipswich, MA, USA	M0205L
Ampure XP	Beckman Coulter	Brea, California, USA	A63881
IDT for Illumina DNA/RNA UD Indexes	Illumina	San Diego, California, USA	20042666

Supplemental Table 5 – Concordance of SNVs and iSNVs between RDTs and PCR swabs

Through analyses limited to only high-quality variant calls and genomic regions with coverage in both samples (Methods), we enumerate the number of SNVs that were detected in person-matched PCR swabs and RDT components. These comparisons are separated into (consensus-level) SNVs and iSNVs (intrahost SNVs). For each comparison, we list the number of mutations present in PCR swabs but not the RDTs (first column), the number of mutations present in both the PCR swab and RDT component (second column), the number of mutations present in the RDT but not the PCR swab (third column), and the percent concordance (fourth column).

	**Mutations present solely in PCR swab**	**Mutations present in both samples**	**Mutations present solely in RDT**	**Percent concordance**
**SNVs from RDT swabs**	0	115	0	100.00%
**SNVs from RDT strips**	0	151	4	97.42%
**SNVs from RDT tubes**	0	19	0	100.00%
**iSNVs from RDT swabs**	6	1	8	6.67%
**iSNVs from RDT strips**	3	0	7	0.00%
**iSNVs from RDT tubes**	0	0	4	0.00%

Supplemental Table 6 – Number of viral reads in samples sequenced via metagenomics

Reads assigned to known, human-infecting viruses via kraken2.

Sample	Description of the sample	Reads assigned to SARS-CoV-2	Reads assigned to influenza A virus (A / California/07/2009 (H1N1))	Reads assigned to human betaherpesvirus 5
M11_swab	positive test Dec 22; putative index case; symptomatic; negative at RDT drive	152078	0	0
M12_swab	positive test Dec 26; symptomatic; negative at RDT drive	22187	0	0
M13_swab	symptomatic; negative at RDT drive	144	0	0
M14_swab	symptomatic; negative at RDT drive	25	0	0
M15_swab	symptomatic; negative at RDT drive	25	1	0
H2O_1	DNase control	2	0	1
H2O_2	DNase control	0	0	0
H2O_3	cDNA control	0	0	0
H2O_E	extraction control	1	0	0
H2O_LC	library construction control	3	0	0

Supplemental Methods

Manual extraction: RNA was extracted from samples using the QIAamp Viral RNA Mini Kit per manufacturer's instructions, with the following changes: i) carrier RNA was not added to AVL; ii) sample input was 700 µl of eluted material; and iii) the 10-minute inactivation step was skipped, as inactivation occurred during elution into AVL. Extraction was conducted by centrifugation or vacuum. After extraction, samples were treated with TURBO DNase followed by a 2X SPRI with RNAClean XP beads.

RT-qPCR: Ct values were derived from RT-qPCR on the QuantStudio 6 Flex Real-Time PCR System with 40 cycles of amplification. We used primers and probes targeting the N1 gene from the SARS-CoV-2 Research Use Only qPCR Primer & Probe Kit as well as the TaqPath 1-Step RT-qPCR Master Mix.

Amplicon-based sequencing: https://benchling.com/s/prt-R95g0tCxKOeCAqn8lAk3. Briefly, we used ARTIC v4.1 primers to prepare Illumina DNA Prep sequencing libraries, which were sequenced on a NextSeq 550. These samples were sequenced in a single batch containing no other samples.

Metagenomic sequencing: Briefly, DNase treatment was followed by cDNA synthesis with random hexamers. Illumina Nextera XT DNA libraries were prepared. Libraries were pooled and subject to target enrichment with the Twist Bioscience Respiratory Viral Research Panel according to the Twist Fast Hybridization Target Enrichment workflow (https://www.twistbioscience.com/sites/default/files/resources/2022-02/Protocol_NGS_TargetEnrichmentFastHybridizationProtocol_2FEB22_Rev4.0.pdf), with a 4-hour hybridization incubation at 60 °C followed by a 14-cycle amplification PCR. Libraries were sequenced with a MiSeq Reagent Kit v2.

Metagenomic assembly: We used Kraken2 with the PlusPF database (downloaded 2022-12-13; https://benlangmead.github.io/aws-indexes/k2) for taxonomic classification. We required samples to have at least 5 reads assigned to a known human-infecting virus, with at least twice as many reads assigned to the virus as assigned to the virus in any negative control. For each virus identified, we conducted *de novo* genome assembly, calling a virus present if a genome of at least 10% of the virus' RefSeq genome length was assembled.

Outbreaker2: Outbreaker2 approximates a posterior distribution of transmission histories via a Bayesian Markov Chain Monte Carlo framework, with data inputs of pathogen genetic sequences (with masking of positions lacking coverage in one or more genomes) and dates of symptom onset. It requires prior distributions for the generation time and incubation duration. Its likelihood consists of 3 components: (1) the genetic likelihood, modeling the probability of observing particular genetic distances, (2) the temporal likelihood, modeling the probability of observing particular symptom onset dates given the generation time and incubation duration distributions, and (3) the reporting likelihood, modeling the probability of unobserved intermediate cases. We used genomic, contact tracing, and symptom onset (or test date, when symptom onset was unknown) data in our reconstruction. We parameterized the generation interval and incubation period as Gamma distributions with means of 2.9 and 3 days, and

standard deviations of 1.6 and 1.5 days, respectively. We performed 100,000 iterations of MCMC separately for samples of each lineage and discarded the first 10% of states each as burn-in. Transmission links with less than 25% probability were excluded from the final reconstruction. To assess the degree to which our results were dependent on our choice of priors, we repeated the reconstruction while toggling the following parameters: setting initial values between 1×10^{-6} and 4×10^{-6} for the estimated per-nucleotide, per-replication mutation rate; testing fixed values of 0.1, 0.75, and 1 as the case reporting probability; enabling the estimation of additional importations; and setting the initial tree topology to a random or star-shaped topology. The reconstruction topologies did not change, with posterior probabilities changing by < 5%.

Supplemental Document 1 – Instructions sent to individuals in cohort 1
6 sets of instructions were sent to individuals in cohort 1, detailing how to take tests, and the order in which to take them.

Instructions (BinaxNOW and Quidel QuickVue)

Introduction: Thank you for participating in our study. In the test kit you have just received, you will find **two rapid tests** for SARS-CoV-2 and **one plastic swab**.

We will try to sequence the genome of the virus that has infected you. Our goal is to compare tests to figure out if there is one that works best in the sequencing process.

We request that upon receipt of these tests, you take all of them **in the order specified below, one immediately after another**. Please **fill out the corresponding blanks** on this form, to be returned alongside the test kits.

Wash your hands

First Test: BinaxNOW

1. **Follow the attached instructions to take the test**
2. **Please fill out the following information:**
   Date & time of test: ___________________
   Indicate the result of the test: ☐ Positive ☐ Negative
3. **Instructions for sending:**
   Leave the cotton swab attached to the test card, then place it in the large plastic bag that the test arrived in.

Second Test: Quidel QuickVue

1. **Follow the attached instructions to take the test**
2. **Please fill out the following information:**
   Date & time of test: ___________________
   Indicate the result of the test: ☐ Positive ☐ Negative
3. **Instructions for sending:**
   Place the test strip in the large plastic bag that the test arrived in, and the tube (firmly capped) in an empty small, rigid bag.

Third Test: Plastic Swab

1. **Follow the attached instructions to take the test**
2. **Please fill out the following information:**
   Date & time of test: ___________________
3. **Instructions for sending:**
   Secure the swab in the collection tube, and place it in an empty small, rigid bag.

Pack Tests into Box and Send via FedEx

Please follow the instructions attached in the following pages.

Instructions (Quidel QuickVue and BinaxNOW)

Introduction: Thank you for taking the time to be a part of our study. In the test kit you have just received, you will find **two rapid tests** for SARS-CoV-2 and **one plastic swab**.

We will try to sequence the genome of the virus that has infected you. This is a scientific process that helps us learn what mutations the virus has. Our goal is to compare these tests to each other, to figure out if there is a test that works best for the sequencing process.

We request that upon receipt of these tests, you take all of them **in the order specified below, one immediately after another**. Please **fill out the corresponding blanks** on this form, to be returned alongside the test kits.

Wash your hands.

First Test: Quidel QuickVue

1. **Follow the attached instructions to take the test**
2. **Please fill out the following information:**
   Date & time of test: ____________________
   Indicate the result of the test: ☐ Positive ☐ Negative
3. **Instructions for sending:**
   Place the test strip in the large plastic bag that the test arrived in, and the tube (firmly capped) in an empty small, rigid bag.

Second Test: BinaxNOW

1. **Follow the attached instructions to take the test**
2. **Please fill out the following information:**
   Date & time of test: ____________________
   Indicate the result of the test: ☐ Positive ☐ Negative
3. **Instructions for sending:**
   Leave the cotton swab attached to the test card, then place it in the large plastic bag that the test arrived in.

Third Test: Plastic Swab

1. **Follow the attached instructions to take the test**
2. **Please fill out the following information:**
   Date & time of test: ____________________
3. **Instructions for sending:**
   Secure the swab in the collection tube, and place it in an empty small, rigid bag.

Pack Tests into Box and Send via FedEx

Please follow the instructions attached in the following pages.

Instructions (iHealth and BinaxNOW)

Introduction: Thank you for taking the time to be a part of our study. In the test kit you have just received, you will find **two rapid tests** for SARS-CoV-2 and **one plastic swab**.

We will try to sequence the genome of the virus that has infected you. This is a scientific process that helps us learn what mutations the virus has. Our goal is to compare these tests to each other, to figure out if there is a test that works best for the sequencing process.

We request that upon receipt of these tests, you take all of them **in the order specified below, one immediately after another**. Please **fill out the corresponding blanks** on this form, to be returned alongside the test kits.

Wash your hands.

First Test: iHealth

1. **Follow the attached instructions to take the test**
2. **Please fill out the following information:**
   Date & time of test: ___________________
   Indicate the result of the test: ☐ Positive ☐ Negative
3. **Instructions for sending**: Please return the test card in a small, rigid bag.

Second Test: BinaxNOW

1. **Follow the attached instructions to take the test**
2. **Please fill out the following information:**
   Date & time of test: ___________________
   Indicate the result of the test: ☐ Positive ☐ Negative
3. **Instructions for sending:**
   Leave the cotton swab attached to the test card, then place it in the large plastic bag that the test arrived in.

Third Test: Plastic Swab

1. **Follow the attached instructions to take the test**
2. **Please fill out the following information:**
   Date & time of test: ___________________
3. **Instructions for sending:**
   Secure the swab in the collection tube, and place it in an empty small, rigid bag.

Pack Tests into Box and Send via FedEx

Please follow the instructions attached in the following pages.

Instructions (BinaxNOW and iHealth)

Introduction: Thank you for taking the time to be a part of our study. In the test kit you have just received, you will find **two rapid tests** for SARS-CoV-2 and **one plastic swab**.

We will try to sequence the genome of the virus that has infected you. This is a scientific process that helps us learn what mutations the virus has. Our goal is to compare these tests to each other, to figure out if there is a test that works best for the sequencing process.

We request that upon receipt of these tests, you take all of them **in the order specified below, one immediately after another**. Please **fill out the corresponding blanks** on this form, to be returned alongside the test kits.

Wash your hands.

First Test: BinaxNOW

. **Follow the attached instructions to take the test**

1. **Follow the attached instructions to take the test**
2. **Please fill out the following information:**
   Date & time of test: ___________________
   Indicate the result of the test: ☐ Positive ☐ Negative
3. **Instructions for sending:**
   Leave the cotton swab attached to the test card, then place it in the large plastic bag that the test arrived in.

Second Test: iHealth

1. **Follow the attached instructions to take the test**
2. **Please fill out the following information:**
   Date & time of test: ___________________
   Indicate the result of the test: ☐ Positive ☐ Negative
3. **Instructions for sending**: Please return the test card in a small, rigid bag.

Third Test: Plastic Swab

1. **Follow the attached instructions to take the test**
2. **Please fill out the following information:**
   Date & time of test: ___________________
3. **Instructions for sending:**
   Secure the swab in the collection tube, and place it in an empty small, rigid bag.

Pack Tests into Box and Send via FedEx

Please follow the instructions attached in the following pages.

Instructions (Quidel QuickVue and iHealth)

1. **Introduction**: Thank you for taking the time to be a part of our study. In the test kit you have just received, you will find **two rapid tests** for SARS-CoV-2 and **one plastic swab**.

   We will try to sequence the genome of the virus that has infected you. This is a scientific process that helps us learn what mutations the virus has. Our goal is to compare these tests to each other, to figure out if there is a test that works best for the sequencing process.

   We request that upon receipt of these tests, you take all of them **in the order specified below, one immediately after another**. Please **fill out the corresponding blanks** on this form, to be returned alongside the test kits.

Wash your hands.

First Test: Quidel QuickVue

1. **Follow the attached instructions to take the test**
2. **Please fill out the following information:**
   Date & time of test: ___________________

   Indicate the result of the test: ☐ Positive ☐ Negative
3. **Instructions for sending:**
   Place the test strip in the large plastic bag that the test arrived in, and the tube (firmly capped) in an empty small, rigid bag.

Second Test: iHealth

1. **Follow the attached instructions to take the test**
2. **Please fill out the following information:**
   Date & time of test: ___________________

   Indicate the result of the test: ☐ Positive ☐ Negative
3. **Instructions for sending**: Please return the test card in a small, rigid bag.

Third Test: Plastic Swab

1. **Follow the attached instructions to take the test**
2. **Please fill out the following information:**
   Date & time of test: ___________________
3. **Instructions for sending:**
   Secure the swab in the collection tube, and place it in an empty small, rigid bag.

Pack Tests into Box and Send via FedEx

Please follow the instructions attached in the following pages.

Instructions (iHealth and Quidel QuickVue)

Introduction: Thank you for taking the time to be a part of our study. In the test kit you have just received, you will find **two rapid tests** for SARS-CoV-2 and **one plastic swab**.

We will try to sequence the genome of the virus that has infected you. This is a scientific process that helps us learn what mutations the virus has. Our goal is to compare these tests to each other, to figure out if there is a test that works best for the sequencing process.

We request that upon receipt of these tests, you take all of them **in the order specified below, one immediately after another**. Please **fill out the corresponding blanks** on this form, to be returned alongside the test kits.

Wash your hands.

First Test: iHealth

1. **Follow the attached instructions to take the test**
2. **Please fill out the following information:**
   Date & time of test: ____________________
   Indicate the result of the test: ☐ Positive ☐ Negative
3. **Instructions for sending**: Please return the test card in a small, rigid bag.

Second Test: Quidel QuickVue

1. **Follow the attached instructions to take the test**
2. **Please fill out the following information:**
   Date & time of test: ____________________
   Indicate the result of the test: ☐ Positive ☐ Negative
3. **Instructions for sending:**
   Place the test strip in the large plastic bag that the test arrived in, and the tube (firmly capped) in an empty small, rigid bag.

Third Test: Plastic Swab

1. **Follow the attached instructions to take the test**
2. **Please fill out the following information:**
   Date & time of test: ____________________
3. **Instructions for sending:**
   Secure the swab in the collection tube, and place it in an empty small, rigid bag.

Pack Tests into Box and Send via FedEx

Please follow the instructions attached in the following pages.

Supplemental Document 2 – Questionnaire given verbally to individuals in cohort 2

Copy of the questionnaire used by MPHN to interview SARS-CoV-2-positive individuals.

INTRODUCTION AND CONFIRMING IDENTITY

Hello, my name is ________. I am calling from the Fitchburg Health Department. We are calling individuals who have recently been tested for COVID-19. Is it okay to continue this call in English, or do you prefer another language?

Out of respect for your privacy, I need to confirm that I am speaking to the right person since we will be discussing health information that is private. Can I confirm that I am speaking with [insert name]? Is that the name you would like us to use on this call?

I see in your COVID testing record that you were born in [insert MONTH only of date of birth]. Can you confirm the date and the year? Thank you for confirming that. Can you also please confirm your current address in (TOWN)?

As you may already know, your COVID-19 test result came back positive, meaning that you are infected with COVID-19. In Massachusetts, certain infectious diseases like COVID-19 are reported to the local health department. We then complete follow-up to help prevent the spread of disease, in this case COVID-19, and provide support, which is the reason I am calling you. Are you available to answer some questions for me?

Have you already spoken with a doctor or a nurse about your test results?

SYMPTOMS

How are you doing/how are you feeling?

Have you had any symptoms? *If yes:* When was the first day you felt any symptoms at all? What symptoms have you had?

Home Isolation Instructions

Let's go over the instructions to safely isolate. We will email you these instructions as well after our phone call, but let's talk about them as well in case you have any questions.

The isolation period for individuals with COVID-19 is at least 5 days from your symptom onset date if you have symptoms, or at least 5 days from your test date if you do not have symptoms. If you are severely ill with COVID-19 or are immunocompromised, the CDC recommends an isolation period of at least 10 and up to 20 days. You should consult with your healthcare provider about when you can resume being around other people.

You can clear isolation after 5 full days if you are fever-free for 24 hours without the use of fever-reducing medication (such as Tylenol) and your other symptoms have improved.

The reason why the requirement is not for symptoms to have fully resolved is because there are some symptoms that may last beyond the previously required 10 day isolation period. An example of this is loss of taste or loss of smell.

If you continue to have a fever or your other symptoms have not improved after 5 days of isolation, you should wait to end your isolation until you meet these criteria. In other words, if you meet these criteria on day 6, day 7, day 8, etc., then you may clear isolation and follow the below guidelines.

If you meet the criteria to clear isolation after day 5, you should:

Wear a well-fitting, high quality mask (such as a surgical mask or a KN95) around others both at home and in public for 5 additional days (day 6 through day 10).

If the case is unable to wear a mask both at home and in public for 5 additional days (for example, a toddler), the case should continue to isolate for a full 10 days.

Avoid travel until 10 days past the date that your symptoms began, or your test date if you do not have any symptoms

Avoid going to places where you are unable to wear a mask, such as a restaurant, until 10 days past the date that your symptoms began, or your test date if you do not have any symptoms

Avoid eating around others at home and at work until 10 days past the date that your symptoms began, or your test date if you do not have any symptoms

If you have access to a test and want to test, you may test with an antigen test (which is what a home test is) at day 5 of your isolation period. You should only test if you meet the criteria we just talked about to clear isolation.

If you do test and the result is positive, this is an indicator that you are likely still infectious and therefore able to transmit the virus to others. You should therefore continue to isolate until 10 days past the date that your symptoms began, or your test date if you do not have any symptoms. If you do test and the result is negative, you should still follow the guidelines that we just discussed, including masking, avoiding travel, etc.

These are things you can do to keep others safe:

Do not leave your home except for urgent medical care. If you must leave, wear a mask as available. Make sure to call the provider before you go and tell them you are diagnosed with COVID-19. Do not take public transportation, ride shares, or taxis.

If you have a mask, you should wear it at all times when around other people, but you should try your best to isolate during your isolation period.

Anyone you come in contact with in your household should wash their hands often and wear a mask if possible whenever they are in close contact with you.

Do not have visitors in your home.

You will need to sleep alone in a separate room if possible.

If possible, use a separate bathroom.

Cover your mouth with tissue when coughing or sneezing and throw the tissue away

Wash your hands frequently throughout the day with soap and water for at least 20 seconds.

Wipe down surfaces that you touch frequently with disposable cloths using bleach if possible or household cleaners.

During your isolation period, if you experience worsening symptoms or have any medical questions, it is important to contact your primary care provider. If you do not have a primary care provider, we can try to help you find one.

If you need help after this call with getting the things you need to stay home safely or need help getting connected to health care or other services, please call this number and we will try to connect you with resources.

CLINICAL INFORMATION
Have you been hospitalized during your illness? *If yes:* What date were you admitted to the hospital? What is the name of the hospital you are/were admitted at?

Do you have any underlying health conditions?

VACCINATION
Now I'd like to ask a couple questions about your COVID-19 vaccination status. Have you been vaccinated against COVID-19?

EXPOSURE INFORMATION
I also have a few questions about where you might have been exposed. Exposure to the virus is often 2-5 days before you developed symptoms.

Did you have a known exposure to COVID-19? If yes: What is the date(s) that you were exposed?

In the week before your symptoms started:

1. Household: Has anyone in your household recently had any symptoms or tested positive?
2. Workplace/School/Daycare/Camp: Have you been to work/school/camp/daycare?
3. Social Gatherings/Events: Have you been to any gatherings or events lately like a concert, birthday party, or wedding?
4. Places of Worship: Have you been to church?
5. Gym: Have you been to the gym?

Cluster specific questions

1. Did you attend the recent craft event in the community room?
2. Do you play cards in the building with other residents?
3. Do you attend bingo?
4. Do you have any friends in the building that you regularly see/spend time with who recently tested positive or have been ill?
5. Are there any other interactions you have recently had with other residents? Carpooling, etc.?

RESOURCES

Next, I'd like to talk about helping you and your household have everything that you need, both while you are at home during your illness and also beyond that. If there is anything that you or your household need, our Care Resource Coordinator will try to connect you to help and support within your community. I'm going to run through some questions to help determine if you have any needs:

Are you worried that now or in the future you will lose your housing?

Are you worried that now or in the future you won't have enough food for you and your family?

Are you worried that now or in the future any of your utilities such as electricity or gas may be shut off?

Do you find it difficult to pay for the basics such as food, housing, medical care, and heating?

Do you feel physically and emotionally safe in your home?

Do problems getting childcare make it difficult for you to work?

Do you have a primary care doctor?

Do you have health insurance?

Do you put off going to the doctor because you don't have transportation?

Do you currently have access to the internet in your home?

If for any reason you need help with day-to-day activities such as bathing, preparing meals, or shopping, do you have the help you need?

If yes: What is the size of your household that would need assistance?

Since you are COVID positive, I would like to share some information about some treatments that may reduce your chances of getting sicker or being hospitalized. These treatments are all great options for high-risk patients who have tested positive for COVID-19. Healthcare providers can determine the need for treatment and prescribe these treatments to eligible patients. This

treatment is not a substitute for vaccination. Everyone is still encouraged to get vaccinated as soon as they can.

Two treatments that are available are called Paxlovid and Molnupiravir. Both are pills taken by mouth and need to be administered within 5 days of symptom onset. Both Paxlovid and Molnupiravir are a 5 day treatment course. Paxlovid is available to eligible patients 12 and older and Molnupiravir is available to eligible patients 18 and older.

The fourth treatment that is available is Remdesivir which is administered through an IV in about one hour blocks for 3 consecutive days. Remdesivir is for eligible patients aged 12 and older.

If you are interested, are you able to discuss these treatment options with your primary care doctor? We also have treatment self-referral options available. UMass Memorial in Worcester has an online form that you can submit and you will be contacted based on treatment availability. The link for this self-referral will be included in our follow-up email or I could also submit this form on your behalf if you'd prefer. When you are contacted, a healthcare provider will determine your eligibility for treatment and prescribe the treatment for you if deemed necessary. They can prescribe all of the treatments that we just discussed.

CONTACTS

Individuals who you came into close contact with while you were infectious should be notified, and we need your help with this. Contacts should be tested for COVID-19 and be vigilant about any symptoms they may develop. Are you able to notify everyone that you have been around since [two days before symptom onset date, or two days before test date if asymptomatic] that they were exposed to COVID-19 and should get tested?

Individuals who may be high risk for COVID-19 include anyone over the age of 65, anyone who is not up to date on COVID-19 vaccines, or those with certain underlying health conditions. If you have any high risk close contacts that you would like us to reach out to, we are happy to do so.

DEMOGRAPHICS

To finish up our call, we would like to ask about some demographic information. This information is important to help us learn so that we can understand inequities, identify gaps, and improve programs that serve all of our communities.

Our first question is, who is your emergency contact or “next of kin”?

What is your email address? This is how we can send you the resources we spoke about earlier for isolation, testing, and community resources.

Our last question is about your racial/ethnic identity. I am going to read a list - if these options don’t fit, please tell me and I’ll make a note. Would you identify with any of the following?

We would also like to ask about your gender. Again, we collect this data so we can understand Massachusetts residents' needs and provide services, not for any other reason. I'm going to read a list - does one of these categories work for you or would you like me to make a note in your file to make one of these more accurate?

Thank you for your time and for helping to keep your community safe. I hope that you find the resources we have provided helpful and remember that all health related questions should go to your medical provider. You may call us back at 978-602-2356 if you are in need of further assistance or need to update any of the information you have shared.
